# Psychosocial experiences of breast cancer survivors: a meta-review

**DOI:** 10.1007/s11764-023-01336-x

**Published:** 2023-03-01

**Authors:** King R., Stafford L., Butow P., Giunta S., Laidsaar-Powell R.

**Affiliations:** 1 Melbourne School of Psychological Sciences, The University of Melbourne, Melbourne, Australia; 2 Centre for Medical Psychology and Evidence-Based Decision-Making, School of Psychology, The University of Sydney, Sydney, Australia

**Keywords:** Breast cancer, Survivorship, Qualitative, Meta-review, Systematic review

## Abstract

**Purpose:**

Advances in breast cancer care have led to a high rate of survivorship. This meta-review (systematic review of reviews) assesses and synthesises the voluminous qualitative survivorship evidence-base, providing a comprehensive overview of the main themes regarding breast cancer survivorship experiences, and areas requiring further investigation.

**Methods:**

Sixteen breast cancer reviews identified by a previous mixed cancer survivorship meta-review were included, with additional reviews published between 1998 and 2020, and primary papers published after the last comprehensive systematic review between 2018 and 2020, identified via database searches (MEDLINE, Embase, CINAHL, PsycINFO). Quality was assessed using the Joanna Briggs Institute Critical Appraisal Checklist for Systematic Reviews and the CASP (Critical Appraisal Skills Programme Qualitative) checklist for primary studies. A meta-ethnographic approach was used to synthesise data.

**Results:**

Of 1673 review titles retrieved, 9 additional reviews were eligible (25 reviews included in total). Additionally, 76 individual papers were eligible from 2273 unique papers. Reviews and studies commonly focused on specific survivorship groups (including those from ethnic minorities, younger/older, or with metastatic/advanced disease), and topics (including return to work). Eight themes emerged: (1) Ongoing impact and search for normalcy, (2) Uncertainty, (3) Identity: Loss and change, (4) Isolation and being misunderstood, (5) Posttraumatic growth, (6) Return to work, (7) Quality of care, and (8) Support needs and coping strategies.

**Conclusions:**

Breast cancer survivors continue to face challenges and require interventions to address these.

Implications for Cancer Survivors.

Breast cancer survivors may need to prepare for ongoing psychosocial challenges in survivorship and proactively seek support to overcome these.

**Supplementary Information:**

The online version contains supplementary material available at 10.1007/s11764-023-01336-x.

## Introduction

Breast cancer (BC), while highly prevalent globally (highest incidence cancer among women in 159/185 countries) [1], has a high survival rateThe average five-year survival rate in Australia for BC from 2013 to 2017 was 91.5%, compared to 69.7% for all cancer types combined [2]. Cancer survivors are defined as individuals diagnosed with cancer who have completed their initial cancer treatment [3], including those considered cured and those living with ‘advanced’ disease (including locally advanced and metastatic cancer), receiving ongoing care.

BC survivors may experience significant long-term psychosocial effects that impact their quality of life, including fatigue, pain, difficulty sleeping, cognitive challenges, sexual dysfunction, depression, anxiety and fear of recurrence or progression [4–7]. Women with advanced BC may additionally experience feelings of abandonment, isolation, existential distress and a loss of control [4, 8, 9]. Thus, BC survivors may benefit from tailored support and care [4, 8].

**Fig. 1:**
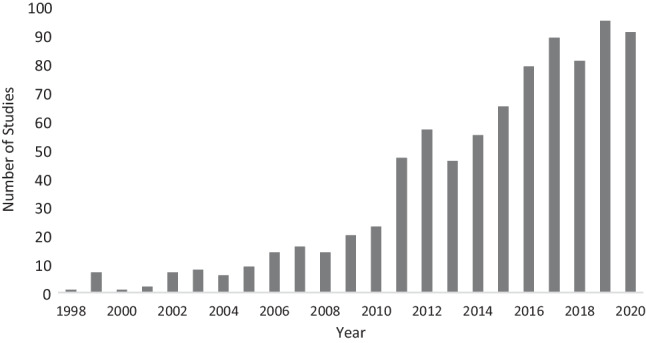
PubMed search results by year for BC survivorship qualitative studies. Articles on PubMed for the search terms (breast cancer) AND (survivor*) AND (qualitative)

To inform development of BC survivorship services, a comprehensive understanding of women’s experiences and needs is required. Qualitative research, which captures women’s lived experiences, is well placed to provide that understanding [10]. The number of qualitative studies focusing on BC survivorship has steadily increased since the late 1990s (see Fig. 1). Systematic reviews which aim to critically appraise and summarise the findings of these primary studies have also increased. While systematic reviews provide a high level of evidence to inform research and policy [11, 12], many BC survivorship systematic reviews focus on specific topics (for example, return to work [13]) or populations (for example, women from a particular ethnicity [14]). To provide a more complete picture of the evidence base and identify areas of research density/paucity, a meta-review (systematic review of systematic reviews) is required. Meta-reviews can synthesise entire fields of research and are particularly beneficial when there is a large evidence base on a topic [12]. Such an overview is required firstly to ensure that new research investigates areas where data are lacking, and secondly to guide BC health professionals in their provision of care, particularly within survivorship centres which focus on this phase of the disease.

To date, one meta-review of qualitative cancer survivorship studies has been conducted [15]. Laidsaar-Powell et al.’s [15] meta-review of 60 qualitative systematic reviews conducted between 1998 and 2018 included 19 reviews addressing BC survivorship. However, due to its wide scope, no specific detail was provided about BC survivorship, and systematic reviews focused on advanced BC were not included. Furthermore, new systematic reviews and primary qualitative studies focused on BC survivorship have been published since 2018. Thus, the current study aimed to conduct an extensivemeta-review of qualitative BC systematic reviews supplemented by a systematic review of primary qualitative studies conducted since the last SR search-date. This BC meta-review aims to provide the first truly comprehensive and exhaustive summary of qualitative evidence focusing on survivorship experiences of women with BC, across both early and advanced stage disease.

## Methods

### Search strategy

This meta-review and systematic review followed Smith et al.’s [12] meta-review guidelines, and adhered to a predefined protocol registered on the International Prospective Register of Systematic Reviews (PROSPERO; registration #CRD42021258728, https://www.crd.york.ac.uk/prospero/display_record.php?ID=CRD42021258728). BC survivorship reviews identified in Laidsaar-Powell et al.’s [15] original meta-review (*n* = 19) were examined for eligibility. Three literature searches were conducted via electronic databases (MEDLINE, Embase, CINAHL, PsycINFO). Search 1 identified systematic reviews published between May 2018 (the latest date searched by Laidsaar-Powell et al. [15]) and August 2020 (the date of this search), used the keywords: (breast cancer) AND (survivor* OR post-treatment) AND (qualitative OR experience OR thematic analysis OR interviews OR focus groups) AND (review OR synthesis OR summary). Search 2 conducted in October 2020, identified systematic reviews focusing on *advanced* BC survivorship. This search did not restrict publication date. It used the same search terms, replacing the keywords: (survivor* OR post-treatment) with (advanced OR metastatic OR late stage). Search 3 conducted in February 2021, focused on primary papers published since the last search conducted for an eligible comprehensive systematic review (January 2018), using the same search terms, but omitting the terms (review OR synthesis OR summary). Search results were imported into Covidence reviewsoftware [16], with duplicates deleted. Study selection, data extraction and bias assessment were undertaken by one author (RK), with 20% independently reviewed by a second reviewer to check accuracy; disagreements were resolved through consensus and discussion with the wider research team.

### Eligibility criteria and study selection

Systematic reviews/papers were included if they were published in English in peer-reviewed journals, and reported qualitative findings relating to BC survivorship experiences. Letters to the editor, conference abstracts, commentaries, and case studies were excluded. Reviews /papers focused on end-of-life/palliative care, caregivers, practitioners, paediatrics/adolescents (i.e. under 18 years of age), or patients currently undergoing or yet to undergo initial treatment, or which focused on service provision, interventions or treatment evaluations, were excluded. Reviews/papers reporting both qualitative and quantitative results or multiple populations (e.g. survivors and patients) were included provided qualitative survivorship findings were reported separately and in sufficient detail to be extracted. Initially, titles and abstracts, then eligible or potentially eligible full-texts, were reviewed for evaluation against eligibility criteria.

### Data extraction and bias assessment

Data were extracted onto a study-designed form (see Tables 1 and 2). The Joanna Briggs Institute Critical Appraisal Checklist for Systematic Reviews and Research Synthesis [11] and the Qualitative Critical Appraisal Skills Programme (CASP) [17] were used for bias assessment (see Supplementary Table 1 and 2 for checklist items). One item for systematic reviews (‘was the likelihood of publication bias assessed?’) was excluded, as it primarily relates to quantitative findings. Each review/paper received a score out of 10 derived from the 10 applicable items (scored as 1 = yes or not applicable, and 0 = no or unclear), with higher scores indicating better quality.

**Table 1:** Included systematic review characteristics and quality ratings

Author Date Country	Aim	Study design	Years of search	Number of primary qualitative studies	Major theme	Summary of qualitative synthesis	Quality rating
AlOmeir et al 2020 [36] UK	Source, appraise, and synthesise data from existing qualitative studies to develop an in depth explanatory model of non-adherence and discontinuation of hormonal medication among BC survivors	Qualitative	2010–2019	24	Health services	Adherence to adjuvant endocrine therapy: --Deciding to start adjuvant endocrine therapy influenced by knowledge, trust in doctors, and worries and expectations --Ability to deal with medication side-effects, knowledge, and support received affect decision to continue; giving up the medication risks cancer recurrence and continuing means reduced contentment --Women stopping medication altogether question treatment necessity, search for normalcy and prioritise quality of life	9/10 (G)
Anbari et al. 2020 [26] USA	Examine and synthesise extant literature that included and assessed the experience of rural BC survivors	Mixed methods	2007–2019	6 qualitative (+ 3 mixed methods)	Rural	Psychosocial support needs for rural BC survivors: --Support networks consisted of family, church family, friends, other survivors, and co-workers --Support from variety of sources could enhance health-related knowledge and behaviours (e.g. using mobile phones or smart devices for communicating with health care providers, accessing education via internet resources, or using social media) Barrier to receiving support: --Lack of transportation was a barrier to accessing survivorship support and education	7/10 (E, F, H)
Armoogum et al 2020 [37] UK	Identify, review and synthesise qualitative research describing the experience of persistent pain in adult cancer survivors	Qualitative	2007–2019	4	Persistent pain	Survivors’ experience of persistent pain: --Interwoven relationship between BC and persistent pain --Lack of preparedness and support for persistent pain --Physical impact of persistent pain --Employing coping strategies --Emotional experience of persistent pain --Conceptualisation of persistent pain	10/10
Banning et al 2011 [13] UK	Examine the qualitative evidence on the lived experience of BC survivors in relation to return to work	Qualitative	1999–2010	10	Return to work	Importance of work: --Returning to normalcy, welcome distraction from cancer, sense of structure, belonging, identity, and social connections Challenges of work: --Treatment-induced physical impairment (e.g. fatigue, lymphoedema), employer comprehension of BC, fear of work-related failure	6/10 (A,C,F,G)
Bettencourt et al 2007 [27] USA	Review the available empirical literature on rural BC treatment and survivorship	Mixed methods	Up to 2006	6 (+ 2 mixed methods)	Rural	Experiences of rural BC survivors: --Those receiving care locally felt more positive than those receiving care further away --Support from members of local community --Coping: avoidance/denial, acceptance, trying to maintain a positive attitude, religious faith	6/10 (A,E,F,G)
Bijker et al 2018 [33] Netherlands	Explore the association between functional impairments and work-related outcomes in BC survivors	Mixed methods	2000–2016	9	Return to work	General Functioning was impaired Physical Functioning: --Problems with mobility and executing physical tasks impacted return to work Cognitive Functioning: --Problems with concentration, attention, memory, pace of thought, multitasking, executive functioning, speed of processing and decision-making Emotional Functioning: --Return to work stressful, frustrating, low-spiritedness, fears, worries, frustrations and insecurity about appearances made it challenging for return to work	10/10
Campbell-Enns & Woodgate 2017 [28] Canada	Summarise the psychosocial experience of women with BC from a lifespan perspective	Qualitative	1990 to year of search	24	Age	Experiences of younger survivors: --Outlook on life changed (life is precious, did not take life for granted) --Gained new sense of mastery --Body changes (menopausal symptoms, scarring) --Fear of cancer recurrence --Impact of cancer diagnosis on family	10/10
Chang et al 2019 [29] Taiwan	Examine the causes of changes in sexual relationships of BC survivors, methods for adaptation to these changes, and health care interventions to facilitate the patients’ return to a normal life	Qualitative		7	Sexual problems	Reasons for sexual relationship changes: --Age (younger experience greater impact) --Treatment Ways that patients adapted to sexual life after the diagnosis of BC: --Support systems --Communication with partners --Religion	7/10 (B, F, H)
Chao et al. 2020 [108] Taiwan	Summarise current knowledge and propose a novel framework for understanding BC survivors’ transition experiences and adopt a more holistic view of transitional care to ensure a successful shift from patient to survivor	Mixed methods	Up to 2018	11	Transition experience	Transition to survivorship involved: --Uncertainty --Fluctuation of medical surveillance responsibilities, feeling adrift from the safety net of the hospital --Relationships changing --Vigilance regarding the recurrence of their BC	8/10 (B, G)
Flanigan et al 2019 [20] USA	Examine how pain and spirituality have been conceptualised, assessed and addressed in women with advanced BC and to evaluate what is known about the impact of spirituality on pain in this population	Mixed methods	2006–2018	7	Metastatic BC	Pain and spirituality in women with advanced BC: --Pain and spirituality as key aspects of illness experience --Fears of a worsening prognosis or impending death --Connection with a higher power and finding meaning in their experience as key mechanisms for facing their advanced BC	9/10 (G) Note: items E and F were not applicable to the scoping review framework of this study
Goncalves et al 2014 [38] Portugal, USA	Highlight what is known about childbearing and parenthood attitudes and decisions of young BC survivors from their own perspective	Mixed methods	1990–2012	5	Fertility	Fertility: --Viewed as secondary to importance of survival/preventing recurrence --Fear of recurrence during/because of pregnancy --Some women desired children in the future, others decided against children due to fears about recurrence/genetic risk/baby’s health	6/10 (A,E,F,G)
Gotay & Muraoka 1998 [115] USA	Review research on the quality of life in long-term cancer survivors to identify quality of life concerns in this population, to provide a critical evaluation of the literature, and to suggest areas for future research	Mixed methods	1980–1998	3	Quality of life/ Symptoms	Women went through many psychological processes to process the cancer experience: --Integrating the disease into current life, confronting mortality, reprioritising, managing changes to relationships, and engaging in problem focused techniques and emotion focused strategies	7/10 (E,F,G)
Henneghan 2016 [35] USA	Explore connections between modifiable factors and cancer-related cognitive impairments in BC survivors who receive chemotherapy as part of their treatment	Mixed methods	2005–2015	5	Cognition	Cognitive changes: --Attributed to lack of mental and physical activity, or lack of social support --Memory dysfunction more pronounced in emotional or stressful situations --Advocated for interventions targeting stress management	8/10 (F,G)
Mollica & Newman 2014 [14] USA	Explore the experience of transition from cancer patient to survivor in African Americans with BC	Mixed methods	1980- 2011	7	Minorities	Challenges of survivorship: --Late side effects, decreased interaction with health care providers, disturbed body image, abandonment or being treated with pity by community Protective factors: Communication with other survivors, spiritual beliefs and connection to church community	7/10 (E,F,G)
Nolan et al 2018 [22] USA	Examine psychosocial concerns in survivorship among young African Americans survivors using a quality of life framework	Mixed methods	2005–2016	7	Minorities	Financial concerns: --Lack of adequate care, medical expenses Sexual concerns: --Impact on sexual desire, relationships, body image, femininity, fertility Cognitive changes Coping strategies: --Social support, spirituality, clinicians	8/10 (A,F)
Russell et al 2008 [23] USA	Gain a better understanding of quality of life in African American women BC survivors. Using Brenner's quality of life proximal–distal continuum	Mixed methods	1998 to year of search	11	Minorities	Challenges: --Physical, psychological, cognitive, sexual, social, financial, existential Coping strategies: --Prayer, avoiding negative people, positive attitude, support from family/friends Cultural challenges: --BC as a ‘white woman’s’ disease, racism, lack of coloured breast prostheses, disparities in support resources	5/10 (A,C,E,F,G)
Selamat et al 2014 [34] Malaysia, Australia	Explore cognitive impairments or chemobrain among BC survivors, with particular attention given to the impact on quality of life	Qualitative	2002–2013	7	Cognition	The chemobrain struggle: --Questioning its existence, feeling of fogginess, attention and concentration difficulties, associated frustration Impact of chemobrain across life domains: --On oneself, social relationships, working life, daily living Struggling to adjust and self-manage: --Lack of support and acknowledgement, development of own strategies Thankful for life, yet fearful for the future: --Concerns about ongoing chemobrain symptoms	10/10
Smit et al 2019 [5] South Africa	Synthesise qualitative studies documenting women's BC narratives into an empirically based explanatory framework	Qualitative	1988–2018	180	Quality of life/Symptoms	Experiences of BC survivors from post-treatment to recurrence and advanced BC: --The burden of BC --Existential ordeal --Illness appraisal --Sources of support --Being in the healthcare system --The self in relation to others --Changes in self-image --Survivor identity	9/10 (G)
Sun et al 2018 [113] Singapore	Explore impact of losing a breast among BC survivors	Qualitative	2000–2015	12	Body image	Body image concerns: --Loss of identity --Changed perception of own femininity --Reduced sexual desire, intimacy, and acts --Isolation from social circle --Perceptions of disfigurement	10/10
Tiedtke et al 2010 [32] Belgium, Netherlands	Explore how female BC patients experience work incapacity during treatment and return to work phases and how interactions between patients and stakeholders affect this experience	Mixed methods	1995- 2008	3	Return to work	Return to work experience: --Disclosure and privacy --Recovery and competence --Attitudes about work --Advice about returning to work lacking --Support and adjustments --Discrimination	5/10 (A,B,E,F,G)
Vivar & McQueen 2005 [30] Spain, UK	Explore informational and emotional needs of long-term BC survivors	Mixed methods	1985–2004	2	Psychological issues	Informational and emotional needs: --Need for more information about survivorship --Fear of recurrence and need for reassurance --Younger survivors needed information about fertility and menopause and experienced sense of isolation	5/10 (A,B,E,F,G)
Wen et al 2014 [24] USA	Review BC experience and survivorship among Asian Americans	Mixed methods	1995–2013	10	Minorities	Importance of culture: --spiritual resources, traditional (Chinese) values, alternative therapy, culture specific support groups Language barriers with health professionals a challenge; low English proficiency Feelings of discrimination in access to quality services Importance of faith/spirituality in coping	7/10 (A,E,F)
Willis et al 2015 [21] Australia	Explore the extent to which the experiences of women with metastatic BC have been investigated, to examine the key foci of such research, and to examine whether there has been exploration of women’s strategies to “live well” with metastatic BC	Mixed methods	1984–2013	14 (+ 2 mixed methods)	Metastatic BC	Experiences of women with metastatic BC: --Living as a social outsider --Importance of hope --Health and quality of life --Positive experiences --Experiences at end of life --Strategies for living	10/10
Yoon et al 2016 [25] USA	Examine factors associated with the quality of life of Korean American cancer survivors	Mixed methods	2000- 2014	5	Minorities	Experience of Korean American BC survivors --Inability to speak English and connect to Western community, difficulty understanding health care system, difficulty in finding an oncologist and support group speaking Korean --Family and others who have experienced cancer main source of support --Challenge-meeting role expectations within family	7/10 (A,E,G)
Zomkowski et al 2017 [31] Brazil	Summarise and systematise the information about physical symptoms and its relation with work activity on female BC survivors	Mixed methods	Up to 2016	5	Return to work	Return to work experience: --Physical symptoms limited work capacity and employability; impacted sleep and driving; lymphoedema distressing and slowed recovery --Little information/advice and difficulty accessing treatment --Fears re capacity, disappointing co-workers, changes/losses in work role --Lack of support from colleagues and employers --Stressful physical jobs more difficult	5/10 (A,F,G,H,J)

*BC* breast cancer. Quality rating score explanation (paper lost marks on the following criteria):

A: Is the review question clearly and explicitly stated?

B: Were the inclusion criteria appropriate for the review question?

C: Was the search strategy appropriate?

D: Were the sources and resources used to search for studies adequate?

E: Were the criteria for appraising studies appropriate?

F: Was critical appraisal conducted by two or more reviewers independently?

G: Were there methods to minimise errors in data extraction?

H: Were the methods used to combine studies appropriate?

I: Were recommendations for policy and/or practice supported by the reported data?

J: Were the specific directives for new research appropriate?

**Table 2:** Included primary papers characteristics and quality ratings

Author Date Country	Aim	Study design	Sample size	Major theme	Summary of qualitative synthesis	Quality rating
Adler et al 2019 [39] USA	Explore the lived experiences of medically underserved women with advanced BC	Qualitative	63	Minorities Metastatic BC	1. Compounding of pre-existing financial distress: Participants experienced financial distress, difficulty meeting basic needs, inability to work, and financil challenges taking precedence over illness concerns 2. Perceived bias/lack of confidence in medical care received: Participants questioned the quality of care they received based on their insurance status and health care settings, and sometimes perceived provider bias related to their low SES and/or race/ethnicity 3. Balancing personal needs with the needs of others: Particpants had increased personal needs, yet often continued in caring roles. Many did not want to burden loved ones and felt grief over increased dependence in daily tasks and financial matters 4. Sources of meaning, coping, and heightened gratefulness: Participants expressed appreciation and enhanced gratitude for the meaningful aspects of their lives, drew on social relationships, spirituality, creative outlets, and altruism to cope	9/10 (F)
Alfieri et al 2021 [40] Italy	Achieve an in-depth understanding of the patients’ needs related to the metastatic BC care pathway	Qualitative	9	Metastatic BC	1. The need for clinical recognition: The metastatic phase unanimously viewed as different from previous phases. Participants reported feeling more emotionally sensitive, more vulnerable, limited in their daily lives, and more encumbered by medical tests and therapies 2. The need for more attention from healthcare professionals: Both from an “information management” point of view and in terms of a more “human” approach 3. The need for more and better services to be available at the hospital: a) better planned services (e.g. need for shorter waiting times) and psycho-social support (e.g. access to psychological and social services, pratcical supports and social/peer group support) 4. The need for specific public health policies: Support with regard to the patient’s rights at work and research into metastatic BC	10/10
Almegewly et al 2019 [130] Saudi Arabia	Explore the experience of being a BC survivor in Saudi Arabia	Qualitative	18	Culture	1. Hidden survival: BC had a cultural stigma linked to death. It changed the sense of self and of society, leading some women to hide their diagnosis from the public and their families 2. The cultural meaning of survival: There was a cultural and religious meaning to survival, linked to God’s will, normality and resumption of activities. Participants rarely used the term ‘survivor’ because it acted as a reminder of an experience that they would rather forget. Others linked being a survivor to returning to normality. Many women called themselves survivors from a physical perspective, but not psychologically, due to the ongoing psycho-social consequences of BC, such as fear of cancer recurrence, lack of support, and disturbance of the marital relationship	10/10
Anbari et al 2019 [70] USA	Examine perspectives of women with newly diagnosed BC-related lymphoedema (BCRL) regarding their quality of life over seven years	Qualitative	97	Lymphoedema	Three major themes were identified related to BCRL’s impact on: 1. Physical function: Pain, fatigue, and being less active (and having to limit or change their routine) 2. Daily living and social function: Limited in jobs and roles (e.g. fustration of not being able to do what they used to be able to do, such as shopping, cleaning), and body image concerns 3. Psychological function: Frustration, depression, and being more irritable	9/10 (F)
Azulay Chertok et al 2020 [84] USA, Canada	Explore the infant feeding experiences of mothers with a BC history	Qualitative	20	Infant feeding	1. Miracle of motherhood after BC: Pregnancy returned a sense of normalcy and control over their bodies that cancer had stolen from them. The ability to breastfeed after cancer seemed particularly miraculous, leaving women feeling accomplished and empowered 2. Medical misinformation or no available information: Contributed to the exacerbation of mothers’ worries 3. Post-breast-cancer feeding challenges 4. Desire for infant-feeding support rather than pressure	9/10 (F)
Bilodeau et al 2019 [59] Canada	Describe the return to work journey from the end of BC treatments to return to work	Qualitative	9	Return to work	The first six months after the end of treatment was identified as an “in-between" period, during which participants questioned their ability to return to normal life due to the impact of side effects and a sense of withdrawal from health-care services. A three-stage journey similar to a rite of passage process (Van Gennep, 1969) was observed: 1. BC survivors became aware of feeling on the fringes of the workplace as they awaited return to work 2. During that waiting period, BC survivors were rebuilding a “normal routine” and taking actions on their own in order to re enter their workplace 3. After return to work, they needed to make adjustments to maintain a work routine	9/10 (F)
Bilodeau et al 2019 [60] Canada	Outline gaps and delay in survivorship care in the return to work pathway of BC survivors	Qualitative	9	Return to work	There was a paucity of dedicated resources for cancer. Lack of survivorship care plans and information about survivorship (e.g. transition to a new normal, side effects, return to work)	8/10 (D, F)
Binsha Pappachan et al 2020 [112] India	Explore the lived experiences of women who are BC survivors	Qualitative	18	Survivorship experience	1. Diagnosis and treatment of BC impact on survivorship: Ignorance of symptoms of BC so symptoms ignored for too long; changes in physical appearance, e.g. hair loss and weight loss; financial burden of cancer treatment, family, work and financial problems 2. Support system impact on survivorship: Support received during and after treatment was key factor for successful completion of cancer journey 3. Issues in survivor life: A tough journey, unsure of the future, fear of recurrence, ready to face anything that comes their way, happy to come back to normal life	9/10 (F)
Black et al 2020 [51] USA	Examine the unmet sexual and reproductive health needs of BC survivors, as well as concordances and discordances in needs by childbearing status and race	Qualitative	17	Sexual and reproductive health Age—young	Four themes highlighting the spectrum of un/met sexual and reproductive health needs identified by BC survivors during their post-diagnosis reproductive years. Despite commonalities, there were differences between women who did have/ desired children and did not have a child after their BC diagnosis, and between women of colour and White women 1. Limited reproductive health information received from cancer care providers 2. Women desired realistic expectations of conceiving post-treatment 3. Women struggled with adjusting to altered physical appearance 4. Menopause symptoms led to sexual health and quality of life issues	10/10
Bolton and Isaacs 2018 [68] Australia	Provide a detailed description of women’s experiences of cancer-related cognitive impairment, its impact on daily life and care received for it following treatment for BC in Australia	Qualitative	50	Cognitive impairment	Experiences of cancer-related cognitive impairment: 1. Difficulty remembering things: e.g. words and spelling, unable to recall faces and names of people they knew well 2. Difficulty recalling previously known tasks: difficulty recalling tasks previously done routinely e.g. where to wash when showering, unable to navigate traffic, could not multitask 3.Inability to stay focussed on a task: unable to complete new tasks, could not focus, easily distracted, going blank, can't stay focused on plot 4. Other symptoms: other discrete symptoms e.g. losing things, difficulty learning new things when this is unusual for them, repeating themselves Impact on daily life: 1. Economic impact: Women unable to return to work and needing to claim social security 2. Psychosocial impact: Reduced confidence in work and social activities, frustration, reduced level of tolerance 3. Minimal impact Care received for cancer-related cognitive impairment: 1. Clinical team's lack of understanding: Poor understanding of symptoms among treating clinicians, no information provided on cognitive impairment, cognitive symptoms are played down 2. Good care received: was well prepared for cognitive symptoms, good support from breast care nurse	9/10 (F)
Brennan et al 2020 [85] Ireland	Explore patients’ rehabilitation experiences and unmet needs during home rehabilitation after BC surgery and to understand their experiences of Mobile health (mHealth)technology and the requirements they desire from an mHealth system	Qualitative	10	Surgery rehabilitation	Rehabilitation experiences: 1. Acute and long-term consequences of surgery: Physical stiffness, musculoskeletal complications, being emotionally unprepared for what to expect 2. Unmet needs and lack of support: Not aware of importance of physio, didn't have enough follow-up with physio, not sure how to do exercises, need more advice/support after discharge 3. Self-driven rehabilitation: Strong motivation to do home rehab, accountability when attending physio as external motivator; stong role of peer support, holistic benefits of exercise 4. Visions for high quality rehabilitation: Patients appreciated proactive care and patient education, recommended providing more info to patients Technology: 1. Experience with digital technology for health: Lack of mHealth options for this clinical context and using non-cancer–specific applications and wearables, technology assisted patient understand information from hospital but cannot replace human health care 2. Requirements for an mHealth system: Requested an mHealth tool from a reliable source to provide exercise support, e.g. information and guidance, audio and visual exercise feedback, less-is-more approach with life affirming content	9/10 (F)
Brown & McElroy 2018 [83] USA	Explore sources of stress and support experienced by sexual and genderminority (SGM) BC survivors and the impact of treatment on their lives	Qualitative	67	Sexual and gender minority	1. Self-disclosure of sexual orientation and gender identity to providers: Not all survivors felt comfortable disclosing their SOGI or identifying their same-sex partner to providers, anticipated negative reactions 2. Need for recognition and support of partners: Those who did disclose were pleased with how their providers treated their partners 3. Experiences in non-LGBT support groups: Mixed experiences. Positive experiences related to the presence of at least one other SGM group member. Benefits included: shared experiences with BC or emotional issues. Participants reported discomfort talking about impacts of BC on their relationships, or felt uncomfortable disclosing their SOGI. Some participants found non-LGBT support groups to be so heterocentric that they felt unwelcome. Non-LGBT-specific support groups were most useful when content was relevant to SGM survivors 4. Experiences in LGBT-specific support groups: Majority did not have access to LGBTspecific support groups for BC survivors in their local area. Not having access made their cancer experience more difficult. Online sources of support have been helpful 5. Impact of treatment on relationships: The most frequently reported long-term impact of BC treatment, particularly for participants taking estrogen blockers or aromatase inhibitors, was the devastating impact on their relationships including sexual intimacy	9/10 (F)
Canzona et al 2019 [77] USA	Identify sources of uncertainty BC survivors and partners of BC survivors report as a result of sexual health changes after primary treatment and to investigate the challenges they experience when attempting to communicate about sexual health-related uncertainty	Qualitative	40 (and 13 male partners)	Sexual health	Sources of uncertainty for BCS and partners: 1. Perceptions of post-treatment body: For BCS: concerns about attractiveness and body image. For partners: Concern about partner's body image concerns and comfort with nudity 2. Worry about effects on relational partners: For BCS: worry that partner feels rejected or dissatisfied. For partners: concern partner's lack of sexual desire is their fault 3. Ethical concerns about dissatisfaction with sexual relationship: (partners only): feeling guilty about dissatisfaction with sex life 4. Fears about future of the relationship 5. Apprehension about SH treatment futility: concern about lack of existing treatment to alleviate survivors’ symptoms Communciation challenges for couples: 1. Supporting survivors’ body esteem: (partners only): Deciding how to respond to survivor silences and/or nonverbal cues, 2. Navigating potentially hurtful disclosures: For BCS: Mistrusting partner assurances regarding appearance/sexual relationship. For partners: Disclosing their thoughts about changes to women’s bodies, Disclosing frustration about their need for sex 3. Responding to partners’ obstructive behavior: (BCS only): Partner avoidance/topic change, Partner defensiveness, Partner unwillingness to seek help 4. Believing communication is futile: (both partner and BCS): No existing treatment to alleviate survivors’ symptoms and prior attempts at communication made things worse	9/10 (F)
Caron et al 2018 [61] Canada	Describe the perceptions of BC survivors on the practices put in place by their supervisors to support them during their return-to-work process	Qualitative	10	Return to work	Three practices helpful during the return-to- work process: 1. Maintaining communication during their period of absence 2. Working with them to structure their return-to-work process before their actual return 3. Allowing them flexibility in their schedule for a certain period Additional: 1. Lack of follow-up over time and this was experienced as a sense of abandonment	9/10 (F)
Ceballos et al 2021 [46] USA	Examine the emotional experience of African American BC survivors, and the information exchange between providers and patients, during transitioning to post-treatment survivorship	Qualitative	45 (and 27 oncology providers)	Emotional needs	Three overarching areas: 1. Emotional impact of transitioning to post-treatment survivorship 2. Types and sources of survivorship information 3. Systemic factors that impact transition to survivorship Participants reported emotional health information and support were needed but not consistently provided, resulting in a sense of survivor isolation	10/10
Chen et al 2020 [106] China	Explore and delineate the dynamic progression toward self-acceptance in Chinese women with BC	Qualitative	20	Self-acceptance	The core category of self-acceptance was normalisation, returning to the pre-illness state with a self-identity and self-image that conformed to the cultural norm. To reach normalisation, women progressed through a crisis stage (characterised by difficulty transitioning from health person to a patient identity; and difficulty accepting a deformed body image), a compromise stage (characterised by passively accepting the 'diseased' identity and gradually compromising with the deformed body image), and a managing impressions stage (characterised by developing practices eg walking, tai chi to recover the healthy identity; avoiding the 'patient' identity, and showing the 'normal' body image to others eg wigs or false breasts). Managing the disease and maintaining a good quality of life after treatment became the women's main goal	10/10
Cheng et al 2018 [105] Singapore	Explore the supportive care needs of women who were in the first 5 years post BC treatment	Qualitative	60	Supportive care need	Five major themes reported by participants: 1. Continuity of care: Participants appreciated cancer specialist care during follow up and surveillance minotoring. Some felt posttreatment physical and psychocosial support and information needs may be unrecognised by health professionals 2. Normalcy and contextual factors: Adaptation to life posttreatment was varied. Some embraced the notion that they were not patients and they were now 'normal'. Family and social networks had a mediating influence on some survivors adaptation 3. Lifestyle advice and self-management: Many participants wanted to be empowered to self manage their own health and wanted information on wellbeing, living a healthy lifestyle including diet, exercise, and breast self‐examination 4. Financial barrier: A few participants expressed finacial issues due to healthcare costs 5. Approaches to posttreatment care: Importance of timely, patient‐friendly language, personalized and specific information to meet their needs for support and information	8/10 (D, F)
Cherif et al 2020 [86] France	Explore BC patients' experiences of healthcare via their online community posts	Qualitative	967 comments on BC forum	Experiences of healthcare	Only survivorship/follow-up theme included in this review: Lexicometric analysis showed a dominance for the word “check-up” associated with medical examinations such as: “mammography”, “radiography”, “ultrasound”, “assessment”, “The anxiety of the biannual check-up after BC”, “from one centre to another the check-up protocols are very variable”. Thematic analysis demonstrates the need for patients to assess the quality of the follow-up and the importance of the performed tests just after the treatments"	7/10 (E, F, G)
Chuang et al 2018 [114] Taiwan	Understand the perception of body from women diagnosed with BC more than 5 years previously and whose treatment included a mastectomy	Qualitative	8	Body image	1. Restoration of the Body Image: Mastectomy changes womens'perceptions of body and provokes a sense of loss of control, as well as self-identity. Many women choose to restore their altered body seeking to regain control over their body, self-identity and a "regular life" 2. Abandonment of Objectification: Women experienced a reduction of both the persistent inspection and evaluation of the body appearance. Women stopped viewing themselves from an observer’s perspective, rather valuing their bodily health, leading to a new value of their body and freedom from objectification 3. Redefinition of Self: The breasts were closely connected with femininity and are related to women’s sense of self-worth. Despite losing a breast/breasts, the women sought to re-establish their sense of self-worth through career and and caregiving achievements. Support from their partners, religion, and other women relieved their distress	10/10
Chumdaeng et al 2020 [81] Thailand	To explore health behaviour changes among Thai survivors of BC following treatment completion	Qualitative	15	Health behaviour	1. Change diets to prevent cancer recurrence: that encompassed having healthy diets to strengthen immunity, and avoiding diets that enhanced the growth of cancer cell 2. do exercise: that involved early arm exercise and regular leg exercise 3. attempt to reduce psychological distress	9/10 (F)
Currin-McCulloch et al 2020 [131] USA	Compare the themes of BC survivorship that emerged from social media data with those that emerged via content analysis of focus group data	Qualitative	1051 original posts and associated comments 18	Survivorship experience	Social media results: (1) diagnosis, (2) social support, (3) risk, (4) existentialism, (5) treatment process, (6) information-seeking and (7) surgery Focus group results: (1) screening/diagnosis, (2) treatment, (3) social support, (4) existentialism, (5) disclosure, (6) coping and (7) fears Comparison between social media and focus group data: Similar themes however the in-person dialogue presented a more nuanced and vulnerable description of the participant’s coping processes, fears of dependency and dying and experiences surrounding disclosure of their diagnosis	10/10
Davis et al 2018 [47] USA	Explore specific patient-centered supportive care factors that were used by African American BC survivors during their survivorship journey	Qualitative	155	Supportive care needs Minorities	Supportive care factors: 1. Faith: Faith in God, attending church, and ongoing prayers, helped coping during survivorship 2. Supportive structures: Support came from family, friends, spouses, and healthcare providers. Many developed some new bonds with other survivors along the illness/wellness trajectory. Participants also said that having health care providers who were caring before, during, and after treatment was “paramount” to their survivorship 3. Optimism: Having optimism and maintaining a positive outlook and a positive attitude during all phases of survivorship were reported as important 4. Access to information: Obtaining cancer information about what was important during medical visits, in treatment compliance, and during the survivorship experience-helped the women during their daily lives	8/10 (E, F)
Dean et al 2019 [71] USA	Compare long-term costs and financial impcacts among women with BC-related lymphoedema to those without a lymphoedema diagnosis	Mixed methods	Qualitative substudy: 40	Lymphoedema	Three main themes provide insight into the burden of higher costs associated with Lymphoedema 1. Economic burden is cumulative and cascades over time: Managing an adverse treatment effect presents ongoing challenges. The use of savings to cover medical costs and additional loans or debt to cover medical costs was common. Women with lymphoedema commonly reported that upfront costs associated set off a cascade of financial challenges that continues to affect their current economic situation 2. Lymphoedema care needs are unlikely to be covered by insurance: Contributes to higher long term costs and compromises a patient’s ability to manage lymphoedema symptoms 3. Productivity losses have long-term impact: BC diagnosis may have influenced work opportunities and long-term earning potential, and BC-related lymphoedema may further decrease productivity losses at work. Women with lymphoedema were less likely to return to employment after cancer because of their additional physical challenges	8/10 (D, F)
Drageset et al 2020 [107] Norway	Describe survivors’ coping experiences 9 years after primary BC surgery	Qualitative	15	Coping	1. Changed Life: Cancer associated changes were challenging, but also enriching. Women had a changed view of life, clarified values and changed priorities in life such as health, family, and close relationships. Certain unexpected changes were difficult to accept, such as reduced energy, less joy of life, and reduced self-esteem 2. Positive thinking- Distancing the negative: The need for positive thinking was important for moving on in life and was also a necessary strategy to keep negative thoughts about cancer and its ailments at a distance. Stratergies to distance negative thoughst included listening to music, hobbies, physical activity, being actively engaged with others, and looking ahead 3. Need for understanding and recognition: Understanding and recognition from family, friends and healthcare providers about their changed life situation were important for all	9/10 (F)
Dsouza et al 2018 [103] India	Explore the experiences and needs of BC survivors in Udupi district, Karnataka	Qualitative	17	Survivorship experience	1. Psychological expressions: The fear of disease, treatment and recurrence was common. Sadness (bejar) was common in all BC survivors 2. Spirituality and misconceptions: God was worshiped after the traumatic experience of getting diagnosed with cancer. At the initial period following diagnosis participants showed resentment and blame towards God. As the disease progressed there was increase in offering prayers and having faith on God. Participants held superstitious beliefs that cancer was caused by a curse from God, curse from elderly people and evil spirits 3. Economic burden: High costs of treatment increased out of pocket expenditure due to lack of health insurance. Some refused the treatment or borrowed money due to cost 4. Body image and bashfulness: Concern about physical appearance, especially hair loss and mastectomy led to change in dressing style. Shyness to expose the parts in front of male doctors for examination. Bashfulness also meant particpants did not share the symptoms with family members 5. Maintaining secrecy: Participants hid their cancer for various reasons including: not wanting their family to be depressed, the hereditary nature of cancer may mean woman are not proposed to as children are more likely to have cancer, concerns people would ask unnecessary questions and misconception of cancer being incurable exacerbating the disease condition leading to bad feelings 6. Needs: Participants expressed needs for a) financial assistance; b) information about post-treatment care such as diet, exercise, and follow-up; 3) family support; 4) counseling to provide moral support and to help think positively	9/10 (F)
Enzler et al 2019 [82] USA	Assess needs of low-income BC patients from patient and provider perspectives	Qualitative	26	Low-income	1. Patient finances a barrier to quality care and a burden on survivors 2. Negative emotions hamper recovery 3. Support groups: These groups help financially and emotionally, but support groups do not help enough, or are not wanted by all 4. The health team is an important source of support: Patients expressed high trust of and gratitude to their health team, however many patients felt information and support provided was not adequate and follow-up appointments were too expensive	9/10 (F)
Faccio et al 2020 [88] Italy	Explore themes of the developmental process of becoming mothers (maternal representations) in three samples of pregnant women: those with a past BC diagnosis, those diagnosed with BC during pregnancy and those without a history of BC	Qualitative	19 (and 19 non BC survivors)	Motherhood	1. Fears and worries: Worried about child's health and development, and their ability to breast feed and its safety. Some also worried about own health and survival. Delicate decision-making weighing up own versus' baby's safety. BC survivors worried re ability to breastfeed and impact on relationship with baby; anxiety and stress due to BC experience meant they approached pregnancy with similar feelings. Healthy women had smaller range of worries focused on health of baby particularly after medical tests 2.Meaning of motherhood: Seen as a great joy and support; BC survivors saw it as an unexpexted gift as previous treatment posed obstacles to becoming pregnant; healthy women saw pregnancy as important life event not preceded by great obstacles 3. Mother-foetus relationship: Women with BC form relationship more on bodily sensations, were less confident about future relationship with child; worried about relationship as will have less time and energy for baby; emphasised support from partner, more focused on support as couple while healthy women focused on triadic relationship, partner as father	10/10
Fassier et al 2018 [62] France	Elicit the needs for return to work after BC and describe the process and the preliminary results of this needs assessment	Qualitative	22	Return to work	Women with BC had different motivations to retrun to work: Related to financial issues, identity, social relationships, perceived utility in society, and the meaning of life. All the women had revised their personal priorities after cancer and wanted a better work–life balance. Most preferred to defer return to work. Immediately post treatment was seen as a time to consider priorities, assess capabilities, adjust post-cancer. Women were at different stages of readiness for return to work	7/10 (D, F, I)
Ford 2019 [48] USA	Describe the stories of African-American BC survivors regarding their journey to successful BC survivorship	Qualitative	12	Minorities	Women reported BC survivorship was a never ending tale 1. Orientation, and then I had cancer: Initial shock and disbelief, was followed by urgent need to get treated, be rid of the cancer and get on with their responsibilities for their family and others, no time for lengthy deliberation. Some had body image concerns but most did not have reconstruction 2. A complication, stopping the silence: Cancer regarded as a death sentence, stigma, silence and secrecy following diagnosis. Breaking the stigma and silence was seen as essential to thriving 3. A further complication, treatment: Women experienced distressing side effects. Some were able to inject humour, or turn losses into celebrations 4. Evaluation: Peace in the shadow of the valley of death: Women described diverse coping strategies, including social support, humour, spiritual and religious connection, helping others in similar situations. Some described needing more culturally tailored support groups and resources 5. Resolution: The new normal: Women described changed perspectives, valuing family more, new focus on health and wellbeing, and being positive 6. Coda, I am still here: Survivors attempted to live and have the greatest life they could, as well as to help others to get through cancer	9/10 (F)
Fouladi et al 2018 [78] Iran	Examine the stages that the survivors go through in their sexual life after mastectomy and to determine factors affecting their sexual behaviors following mastectomy	Qualitative	30	Sexual health	Sexual function breakdown: 1. Perceived physical changes: Losing the half of one's body, mutilation, lack of symmetry, becoming crooked, and taking on the appearance of men 2. Altered sexual behaviours: Perceived physical changes and chemotherapy drugs' side effects result in libido decreases and withdrawal from sexual behaviour 3. Factors exacerbating sexual function breakdown: Behaviours and expectations of the sexual partner, the survivor's negative perceived body change, and negative attitudes to the disease in society, pitied by others, with fears of losing her place among her husband's relatives because of her disease Struggling to restore and reconstruct sexual function: 1. Modifying sexual behaviours: Pretending to be engaged and energetic in sexual relations contrary to inner feelings 2. Efforts for physical restoration: Through prosthesis, padded bra, clothing 3. Striving to gain support: Spouse, children, belief and trust in God	6/10 (D, F, G, H)
Ginter 2020 [41] USA	Examine the process of meaning making by young women diagnosed with metastatic BC	Qualitative	9	Metastatic BC Age—young	1. This shouldn’t be happening now: Untimely diagnosis created shock and anger. Treatment side-effects (e.g. menopause) also untimely 2. Strategizing disclosure: Disclosure was difficult because reactions were sombre and fearful, and recipients did not know what to say. Explaining cancer required emotional energy 3. Benefits of mindfulness: Helped distract from fears and cancer concers 4. Contemplating the future: Future planning very difficult. Sense of limited time. Challenges of financial planning and procuring life insurance, knowing when to plan end-of-life issues such as the funeral, existential crisis, fearing being forgotten, increased focus on spirituality, living in the present to avoid thinking about the future 5.Agency in perspective: Sense of empowerment from thinking positively, focusing on not just surviving but living well	9/10 (F)
Gorman et al 2020 [58] USA	Examine how young adult BC survivors and their partners appraise and manage their sexual health and intimate relationships after cancer	Qualitative	25 (and 25 partners)	Sexual Health Age—young	1. Shared understanding of physical and psychological challenges of sexual health after cancer: Often led to reduced sex and breakdown in communication about sex. Women reported guilt. Couples with good communication more likely to come up with creative ways to still enjoy sex 2. Navigating role shifts and changes to sexual relationship: Variation in how women transited from patient role to sexual role. Some couples experienced difficulty, others felt they came together more closely 3.Getting through it as a team: Mutual support was seen as critical 4.Maintaining open communication: Communication was seen as vital but difficult, due to stigma around sex, lack of experience and skills to talk about sexual problems, and heighted emotion 5. Need for services and support for partners/ caregivers and couples: Couples wanted more help, preferred a couple approach	9/10 (F)
Henderson et al 2019 [69] UK, Singapore	Explore the experience of living with chemobrain, within an illness representation framework, in BC patients	Qualitative	12	Cognitive changes	1. The new normal: Participants reported greater effort required in normal tasks, loss of immediate memory and higher order cognitive function. Consequently, women had to adjust to changes in personal identity, social interactions and as adjustment to working life ( a "new normal") 2. Beliefs and expectations: Most women did not improve over time and found disappointed expectations hard to bear. Most ascribed cause of chemobrian to chemotherapy, but were not certain, and felt that other aspects of the cancer experience (e.g. stress) may have contributed. Not helped by insufficient information from health professionals. Women found it helpful to attribute cognitive deficits to chemobrain 3. Coping with chemobrain: Perceived control, planning, preparation and writing things down helped. Some women tried to exercise their brains to foster improvement. People found family, friends and work colleagues supportive, however disliked false reassurance. Embarrassment leading to non-disclosure could limit support. Reciprocity with fellow sufferers helped	9/10 (F)
Henneghan et al 2018 [52] USA	Explore the unique experiences of survivors who were diagnosed and treated for BC, including chemotherapy, while in their 20 s	Qualitative	3	Survivorship experience Age—young	1. Premature family planning: Having to make serious and complex decisions about family planning in the midst of diagnosis; 2. Impact on future planning and outlook: Became more positive, but also had increased fear of recurrence, anxiety, and a "hypochondriac‐like" awareness of their bodies; 3. Lack of confidence: And also feelings of insecurity; different to older women with BC, being young and navigating such a complex health issue was challenging, did not identify with BC, wanted contact with other young women; 4. Chemobrain: Particularly in the work place; 5. Impact on relationships: both positive and negative; sexual and intimacy challenges, and changed experience of stress	4/10 (A, D, E, F, I, J)
Hubbeling et al 2018 [53] Mexico	Describe the psychosocial needs of young BC survivors in Mexico at 5 or more years of survivorship, identifying areas of focus for early interventions	Qualitative	25	Psychosocial needs Age—young	1. Minimisation of fertility concerns: In survivorship some women described regret and difficulty accepting the reality of infertility. Limited discussion with health professionals 2. Persistence of body image disturbance over time: Some described an initial `blow' to their body image followed by slow adaptation, while others denied body image disturbance at any point. Reasons for not undergoing reconstruction after mastectomy included prohibitive expense, risks and uncertainty about how to pursue the procedure. Varied impact on personal and sexual relationships 3. Barriers to employment during survivorship: Need to financially support family. Time occupied by work as taking away parent–child time. Some took time off work due to medical appointments, fatigue, or type of work. Patients who continued work described work as a support, distraction, or source of normalcy. Women who had difficulty finding employment after BC noted a lack of accommodation for absences for medical visits and new physical limitations. Many perceived hiring discrimination leading to fear of disclosure 4. Impact on family relationships and social networks: Concern for children and family members. Physical separation of families resulted in significant strain. Experienced narrowing social circles, social isolation, stigma in the community 5. Unmet psychological care and informational needs: Women had unmet need for psychological care. Felt out of touch with support groups and other programs at the hospital. Those who saw psychiatrists described immense benefit. Barriers to receiving psychological care included cost, time, and uncertainty about how to find a provider	9/10 (F)
Inan & Ustun 2020 [89] Turkey	Explore the nature of posttraumatic growth in Turkish BC survivors in the post-treatment first two years	Qualitative	13	Posttraumatic growth	1. Making sense of the cancer: Questioning life and death and religious meaning 2. Positive restoring: Changes in values of life, self-value and relationships, and increased coping skills	9/10 (F)
Jakobsen et al 2018 [95] Norway	Describe the everyday life in BC survivors experiencing challenges	Qualitative	11	Survivorship experience	1. ‘Bodily and mental loneliness’: *a) Bodily and mental challenges:* physical symptoms (e.g. pain, sleep difficulties, tiredness, cognitive impairment) impacted on BC survivors ability to do things *b) Information and timing mismatch:* Lack of information and difficulties in finding relevant and comprehensible information. To gain access to relevant information, some joined the Breast Cancer Association while others consulted acquaintances or associates, or other people who had experienced cancer *c) Relationship and partnership:* Open communication with loved ones was valued. However, because of reduced physical and mental capacity, some were less socially active. Several BC survivors disliked their bodies, felt unattractive after surgery and wished to have reconstruction to restore their perceived value as a woman. Sexual situations, and especially sexual initiation, were challenging and difficult 2. ‘New centre of gravity in everyday life’ *a) The meaning of work:*work described as meaningful. Many had to make adaptions to work routine/ tasks, however some had faced challenges implementing adaption. To avoid criticism some tried to update their skills. For many, quitting their jobs seemed necessary to preserve their health. Many found other occupations e.g. volunteer or casual work *b) Reorientation of daily occupations:* importance of being physically and mentally active. Appreciated having regularity and routines in their daily life. Someexpressed grief over not being able to engage in the same occupations they previously enjoyed	9/10 (F)
Keesing et al 2018 [96] Australia	Aimed to explore the challenges of women and their partners as they attempted to resume activities and roles, identify unmet needs and make recommendations regarding a suitable framework to support women and partners to recommence valued activities and important roles during early survivorship	Qualitative	18 (and 8 partners), and two focus groups (n = 10)	Survivorship experience	1. Ambiguity regarding survivorship prevents resumption of activities and previous roles: experienced a conflict between their previous identity of cancer ‘patient’ versus ‘survivor’. Ongoing symptoms and adjuvant therapies affected engagement in their usual activities, including difficulty maintaining interests and social commitments. All women agreed that the experience of BC had changed them as a person. Resuming activities disrupted, due to ongoing physical and mental issues 2. BC continues to impact a couples’ relationship during survivorship: Poor communication, loneliness, being unable to resume previously shared interests and responsibilities and feeling overwhelmed when attempting to renegotiate their previously healthy relationship. Concern was raised about changes to body, lack of desire for intimacy and reduced sexual response 3. Support is needed to assist women and partners to resume activities and important roles: All felt a sense of abandonment by the supports and services that assisted them during treatment and felt unprepared for survivorship. Need for survivorship resources for couples	9/10 (F)
Keesing et al 2019 [97] Australia	(1) Determine the physical, psychological, and emotionalneeds of women survivors of BC and their partners and their relation to a returnto previous levels of function, (2) Identify the type and range of current supports provided from the perspective of health providers, and (3) Determine whether the needs of women and partners are adequately met by the existing services provided	Mixed methods	18 women (and 8 partners)	Psychosocial needs	1. Physical needs: Assistance for cancer surveillance, monitoring of medication and side effects. Needing advice regarding consequences of surgery, reconstruction and alternatives, preventative hysterectomy. Managing the long-term effects of BC 2. Psychological/emotional needs: Cognitive (memory and concentration) problems. Sadness, depression, anxiety, mood swings, anger, helplessness, loss of control, frustration and guilt. Fear of cancer recurrence. Impaired body image, disfigurement, feeling less feminine, loss of sensation in breast/s 3. Psychosocial needs: Relationship difficulties, including: communication, intimacy, resuming a sexual relationship with partner. Difficulty resuming daily routines and responsibilities. Spiritual concerns. Resuming friendships and social life. Partners feelings of detachment and isolation, internalizing feelings and difficulty expressing thoughts and feelings	9/10 (F)
Kim et al 2020 [98] South Korea	Explore the self-management needs of BC survivors who had completed treatment	Qualitative	20	Self-management needs	Posttreatment self-management needs of BCSs: 1. Symptom management needs: Need for knowledge and skills to manage persistent symptoms—specifically, participants suffered from gastrointestinal symptoms such as indigestion and appetite loss, peripheral neuropathy, insomnia, fatigue, and sensory problems 2. Emotional management needs: Need for skills to manage emotional problems (fear of recurrence, depression, anxiety). Need for mental health services 3. Information acquisition needs: Need for up-to-date and in-depth information about disease management and lifestyle management 4.Need for a relationship with healthcare providers: Need for patient-centered care, effective communication skills, empowerment 5. Adaptation needs: Need for skills to adapt to changes (body image change, lifestyle change, role change), building Self-confidence	9/10 (F)
Kim et al 2020 [73] South Korea	Identify the experience of BC survivors regarding cancer-related fatigue, exercise and exercise adherence	Qualitative	16	Fatigue, exercise and exercise adherence	1. The insidious and overpowering nature of cancer-related fatigue: Participants reported a high level of cancer-related fatigue after treatment that represented a wide range of both physical and psychological experiences 2.Exercising when experiencing fatigue surrounded by prevailing myths: participants recognised the importance of exercise and strived to increase their physical activity, despite weaker conditions and multiple challenges. Myths/misinformation around exercising 3. Multiple barriers to exercise: Treatment-related side effects, self-image and relationship issues, ambiguous and inadequate information, feeling limited and wary of participating in existing programs 4. Facilitative factors to continue exercising despite fatigue: Comfort in taking care of self. Finding strength through interacting with other BC survivors	9/10 (F)
Knaul et al 2020 [99] Mexico	Explore the concept and experience of survivorship for Mexicans living with breast, cervical, and prostate cancer	Qualitative	22	Survivorship experience	1. Adverse physical and sexual experiences 2. Emotional problems 3. Cancer-related stigma; including return to work 4. Challenges to obtaining health-related information 5. Financial hardship 6. Experience of strengthening family ties in order to provide them with support Additional findings: In addition, women with BC reported distress caused by changes in body image, positive experience with support groups, concerns about the future	9/10 (F)
Lai et al 2019 [90] Taiwan	Explore the experience of recurrence fears among Taiwanese BC survivors	Qualitative	11	Recurrence fears	1. “Trapped in insecurity”: worry about treatment efficacy during period following the completion of primary treatment. Participants felt vulnerable and fearful, feeling trapped in an insecure situation 2. “Suffering in silence”: didnot discuss fear of recurrence with family as it was too stressful or to protect family. Participants tried to keep the news of their cancer diagnosis from others which contributed to a feeling of loneliness and suffering in silence. Concern of cancer recurrence during suvivorship were not empathised by physicians 3. “Pretending as if nothing happened”: The participants attempted to avoid reminders of cancer, they tried to hide or ignore negative feelings. To continue their family role, participants forced themselves to pretend as nothing had happened	10/10
Lambert et al 2018 [93] Canada	The purpose of this study was to explore BC survivors’ experiences and perspectives of persisting with adjuvant endocrine therapy persistence and to identify the psychosocial and healthcare system factors that influence adjuvant endocrine therapy persistence	Qualitative	22	Adjuvant endocrine therapy	The personal, social, and structural factors found to influence adjuvant endocrine therapy persistence included side effects, perception of BC recurrence risk, medication and necessity beliefs, social support, the patient-provider relationship, and the continuity and frequency of follow-up care. For most women, over time, the decision-making process around adjuvant endocrine therapy persistence became a balancing act between quality of life and quantity of life	10/10
Lee Mortensen et al 2018 [42] Denmark	This qualitative study aimed to explore the long-term health-related quality of life and support needs in metastatic BC patients of all ages in the Danish context	Qualitative	18	Metastatic BC	1. Quality of life impact of metastatic BC: *a) reactions to the MBC diagnosis:* shock and fear of imminent death. reduced axiety when treatment initiated. MBC diagnosis marked end to ‘ordinary everyday life'; *b) cognitive:* problems with memory and concentration but none felt this significantly reduced their QoL; *c) physical:* physical QoL and functioning was severely impaired by pain, fatigue and bodily ailments; *d) psychological:* Emotional hardship, depression and anxiety. Living on borrowed time; *e) social/relational QoL aspects of MBC:* children’s welfare, prioritizing meaningful activities and relations; *f) strategies to cope with MBC:* many found that ‘getting things sorted’ – e.g. making arrangements for the funeral – enabled a feeling of control and to focus on living. Maintaining normality was of major importance 2. Treatment and support needs *a) treatment needs:* e.g. improved options of receiving manual physiotherapy to alleviate the physical pain and discomfort; *b) worries related to treatment and controls:*concerns about whether the cancer had spread, about running out of treatment options and the QoL impact of the next treatment; *c) minimizing time spent on treatment; d) socio-economical clarification:* need for a comprehensive approach to MBC care including professional social support. Women with cognitive problems wished for a health care coordinator to help with medical and social aspects of living with MBC; *e) psychological counseling:* Lacking psychological support was the greatest unmet need; *f) information needs:* While most felt adequately informed about standard treatment options, a few called for early genomic testing to prepare for treatment options. Need for psychological support for patients and partners	8/10 (F, G)
Levkovich et al 2019 [74] Israel	Explore the ways in which BC patients, up to a year after the termination of chemotherapy, experience the symptoms of fatigue, how they experience its effect on their lives, quality of life and how they cope with it	Qualitative	13	Cancer-related fatigue	1. “Being imprisoned in the body of an 80-year-old,” the overwhelming effect of fatigue on every aspect of life, and on the women’s quality of life and self-perceptions: Fatigue symptoms, consequences of fatigue including helplessness, lack of control, uncertainty, and emotional distress. Differing fatigue patterns in women, younger women noted reduced caring ability, older women felt like a burden to their families. Strategies for coping with fatigue included task reorganization and reduction, acceptance, and letting go 2. “Family’s bear-hug” exemplifying the role of the familial environment in coping with the experience of fatigue, dealt with the complexity of the women’s interactions with the support systems: Support from community and husbands was helpful. Women had diffculty accepting help. whne support was not provided or was inappropriate to the woman’s needs it lead to hurt, disappointment and loneliness. Women feared loss of privacy and independence	10/10
Liska et al 2020 [100] Canada	Assess the current practice of transition of care following radiation therapy for patients with BC in Alberta, and to identify any additional supports needed to help ease the transition from daily treatments to survivorship	Qualitative	10	Survivorship experience	1. Skin care education: Lack of understanding of how severe the post-treatment side effects could be. Some reached out for further information/supplies, some struggled with self-care, but were reluctant "bother' healthcare team 2. Post treatment phone call: As side effects are at their peak approximately two weeks following radiation therapy, patients expressed that having a follow up phone call during this period would have been a welcome reassurance for them 3. Informational mediums: Prefernce for way to receive information varied. A number preferred email, written format or most commonly, verbally. Particpants were unsure of reliability of information on the internet 4.Returning to normalcy: Return to work support, spirituality and faith, lack of understanding from friends and family 5.Psychosocial issues following treatment: the weeks immediately following the cessation of treatment were emotionally trying. Lonely, missed the sense of connection that they shared with other women they met at the cancer centre. Transitioning from support of healthcare workers was intimidating. Uncertainty. Anxiety and nervousness while awaiting their follow up visit 6. Patient satisfaction with the care provided: Most said they received high quality and personal care regardless of what centre they received treatment at. Patients expressed a strong sense of gratitude towards those involved in their radiation therapy	9/10 (F)
Llewellyn et al 2019 [116] UK	Identify and explore those needs and concerns that have continued to persist, despite advances in treatment and care	Qualitative	13	Psychosocial needs	1. Using personal resources: the ongoing importance of drawing upon intrinsic psychological resources, and how adaptive personal strategies are crucial in ensuring active coping and making sense of one’s experience. Sub-themes: being resilient, seeking a sense of normality, talking about cancer and finding meaning, and coping with an uncertain future (including fear of recurrence) 2. Needing support from others: the desire for high quality communication by healthcare professionals and the importance of ongoing emotional assistance provided by others within and beyond the healthcare system. Sub-themes: needing to feel acknowledged and treated sensitively, managing expectations, needing emotional support, needing to have faith in others, and feeling isolated when treatment ends	9/10 (F)
Lundquist et al 2019 [43] USA	Describe and to interpret the lived experiences of young women with advanced BC from their perspective, as well as to determine what shared experiences exist among this population	Qualitative	12	Metastatic BC Age—young	1. Wearing the mask of wellness in the presence of life-threatening illness 2. Wanting to be known as the person I am: Participants wanted not only to be known as a person with a disease but also as a person apart from the disease 3. I'm still Mom: Desire to continue parenting one's children despite the difficulties and challenges faced as a result of stage III or IV disease 4. Living is more than surviving: Trying to live as fully as possible within the constraints of the disease 5. Getting through it: Coping with advanced BC, including emotional and behavioural components. Maintaining a sense of control and maximising experiences 6. Being connected to others: Need for and challenges regarding relationships with others while living with advanced BC	7/10 (F, G, H)
Luo et al 2019 [63] China	Determine whether BC survivors at work following the diagnosis and/or treatment of BC, in a rapidly developing country such as China experience similar to return to work challenges as reported in nations with established return to work policy and procedures for employees with cancer	Qualitative	16	Return to work	1. Challenges at work related to residual effects of diagnosis and/or primary treatment: Physical limitations, cognitive limitations 2. Positive and negative responses from employers and/or supervisors: Support from employers/supervisors (accommodating efforts, economic support, cohesion, supportive comments), negative reactions from employers/supervisors (bullying, lack of empathy, overprotection, expectations of abrupt return to pre-cancer expectations) 3. Positive and negative responses from co-workers/colleagues: Positive support from co-workers (supportive behaviours, positive feedback—verbal, non-verbal), negative reactions from co-workers (troublesome behaviour, lack of sensitivity). Although several participants experienced a high level of workplace support, there was a subgroup that did report challenges related to symptom burden, cognitive limitations, and both positive and negative responses by employers and co-workers were reported	9/10 (F)
Maheu et al 2019 [91] Canada	Understand the nature of women's cognitive and emotional issues from fear of cancer recurrence using specific guidance from the model by Lee‐Jones and to provide suggestions for modifications to the model based on empirical results from the reported experiences of women living with BC	Qualitative	12	Fear of recurrence	1. Fear of cancer recurrence is always there 2. Beliefs about risk of recurrence: Differing beliefs regarding the risks of cancer recurrence 3. Beliefs about eradication of cancer: Differing beliefs 4. Preferences not to seek information about recurrence: Varied, most participants believed that overwhelming amounts of information and particular types of information such as recurrence statistics could be a source of distress 5. Derailment of normal life: Uncertainty 6. Worries related to recurrence: Worries of reliving cancer, deteriorating, and dying 7. Need for support (beyond treatment): Most women also expressed a strong need for more emotional support from health care professionals, along with support need for follow‐up care guidance and how to manage the late effects of cancer treatment	9/10 (F)
Milosevic et al 2020 [54] Canada	Explore young BC survivors’ beliefs and practices regarding physical activity, healthy eating, and weight management	Qualitative	12	Age—young Physical activity, nutrition and weight management	One overarching theme – An ongoing tug of war – was identified, which reflected participants’ on-going efforts to balance the benefits associated with being physically active, eating healthy, and not drinking alcohol with the challenges associated with maintaining these behaviours: (a) prolonging life with a healthy lifestyle versus enjoying living (b) perceiving benefits versus barriers (c) seeking social connection versus protecting the self from social threats	9/10 (F)
Mosher et al 2018 [44] USA	Identify factors underlying symptomatic metastatic BC patients’ perceptions of the importance of seeing improvement in symptoms following an intervention	Qualitative	25	Metastatic breast BC	Five interrelated factors underlying metastatic breast patients’ perceptions of symptom importance: activity restriction, concentration difficulties, exacerbation of other physical symptoms, symptom-related long-term health concerns, and negative impact on their relationships with others	9/10 (F)
Nolan et al 2019 [49] USA	Describe the lived experience of survivorship among young African American women who completed active (i.e. surgery, chemotherapy, and radiation) treatment for BC	Qualitative	15	Survivorship experience	Participants perceived survivorship as a labile ‘new normal’ and ‘ongoing struggle,’ in which spirituality and survivorship knowledge were key to restructuring their lives 1. Actively managing spiritual self: Hopefulness, life purpose, positive/spiritual change, religious/spiritual activity, uncertainty 2. Actively managing physical self: Nausea, appetite/weight changes, constipation, menstrual change/fertility, sleep, aches/pains, fatigue, skin/hair changes 3. Actively managing psychological self: Anxiety/distress and depression, fear, guilt, cognitive changes, overall perception of quality of life and satisfaction 4. Actively managing social self: Employment and financial burden, social support, personal relationships and role changes, sexuality, isolation 5. Seeking survivorship knowledge: Personal and others’ experiences, healthcare providers and ancillary professional’s expertise	10/10
Oostra et al 2020 [45] USA	Assess nutritional problems and concerns of women with metastatic BC and to explore how to address these problems within an existing eHealth platform	Qualitative	21	Nutritional problems Metastatic BC	1. Knowledge about nutrition 2. Nutrition information-seeking 3. Social aspects of nutrition 4. Nutrition interest 5. How to address nutrition with an eHealth platform	9/10 (F)
Ostby et al 2018 [72] USA	Explore patient perceptions of barriers to self-management of BC -related lymph0edema and gain insight to patient perceptions of BC-related lymph0edema education and support	Qualitative	Focus group (n = 9) and mailed surveys (n = 12)	Lymphoedema	1. Lack of BC-related lymph0edema patient education provided by health care providers: (a) timing and volume of information; and (b) minimalization of BC-related lymphoedema education and inaccurate information from health care providers 2. Lack of understanding by others (Psychological distress): (a) feelings of marginalisation; and (b) non-therapeutic communication 3. Decreased self-efficacy: (a) treatment burden; and (b) lack of follow-up support	9/10 (F)
Pembroke et al 2020 [101] USA	Understand the unmet needs of patients treated with radiation therapy for BC using the five domains of the SUNS instrument as a framework (Hall et al., 2014; Campbell et al., 2010). And evaluate for any area of concern not included in the SUNS categories for survivors of BC after completion of radiation therapy	Qualitative	18 (including 1 male)	Psychosocial needs	Themes emerged from the emotional, relationships, and information needs domains: 1. The struggle with adapting to body image changes 2. Living with the fear of recurrence 3. The unexpected impact of radiation dermatitis 4. The need for education to prepare for radiation therapy	9/10 (F)
Penner et al 2020 [75] USA	Through asking participants to describe their bodily experiences of fatigue- how CRF feels and how they react to those feelings- we hoped to rigorously elucidate trends in the multi- dimensional and highly heterogeneous experience of CRF	Qualitative	13	Cancer-related fatigue	1. The felt experience of cancer related fatigue: “Energy Crashes", ever-Present imbalance 2. Interpersonal influences; clinical and familial: Social perceptions of fatigue, social components of medical systems, cultural values and fatigue. Including sense of guilt 3. Changes in bodily awareness following cancer treatment: Experiences of anxiety, shifts in bodily awareness as a function of fatigue	9/10 (F)
Pintado 2018 [102] Spain	Analyze the meaning of cancer (situational meaning) in a sample of women suffering BC and to relate it to the emotional wellbeing, using mixed method design	Mixed methods	131	Posttraumatic growth	1. A growth in awareness at the present moment: *a) A painful experience*: Some said cancer meant a lot of pain (physical and psychological); *b) The worst of my life*: For 13 women, this pain was the worst situation they had lived; *c) A stop in life's rhythm*: illness helped some women to stop and to observe their lives; *d) A predictable and foreseeable thing*: Six women said that cancer was understood as an expected event due to family history; *e) A growth in knowledge about themselves:* illness meant an increase in self-knowledge; *f) A neutral situation*: For a few women, cancer did not mean anything positive or negative, but a thing that just appears; g*) A bigger faith in God*: For only one woman, cancer increase in her faith in God; *h) A change of perspective and behaviours*: positive change in lifestyle, adopted action based on pleasure and meaning 2. Usefulness of suffering experienced: *a) To stop suffering from insignificant thing*; *b) To value the fight of other patients suffering cancer; c) To be more sensitive and affected:* some were now more worried about their job, money, and future; *d) To gain perspective about myself, knowing my strengths*: cancer allowed discoveries about their own strengths; *e)To be more compassionate and altruistic*; *f) It has not been useful*: Twenty-nine women said that cancer and suffering was not useful for anything; *g)To know how people love me:* For one patient, cancer had meant greater awareness of how people love her	9/10 (F)
Rafn et al 2020 [87] Canada	Explore the experiences among BC survivors, rehabilitation professionals and surgeons on current rehabilitation services within the public setting	Qualitative	35	Survivorship experience	1. Cut the cancer out and goodbye: No or insufficient patient education, Lack of (awareness of) public rehabilitation services, Insufficient training of healthcare professionals 2. You have to look out for yourself: Worry and uncertainty about solo management, People with resources can have the services 3. In a perfect world: learning from the good example, Multimodal pre-surgery education and post-surgery follow-up, Varying perceptions on self-managed surveillance for upper body issues	9/10 (F)
Rapport et al 2018 [109] UK	A. Identify how risk is defined by women, at various stages of investigation, diagnosis, treatment, and care for BC, leading up to remission B. Describe the different journeys women take along the care continuum, and their own expressions of need and experience as they move through the health care system toward better health C. Disclose the views of a wide range of female patients, from those undergoing genetic investigation for risk of BC to those undergoing post BC treatment	Qualitative	25	Survivorship experience	1. Subjective Understandings of Risk: *a) Risk assessment, risk status, and risk classification*—facts around risk that related to a woman’s own concerns were overshadowed by others’ agendas; *b) Impact of risk on family members and close others*—conern about the negative consequences of risk for other family members; *c) Personal understandings of risk* – risk was a sense of “being” or “knowing” one’s body differently; *d) Sharing information through health care professional involvement:* shock following diagnosis prohibited women from fully absorbing all the information from oncologists. Service provision following risk assessment 2. Journeying Toward an Unknown Future: *a)Undetermined consequences of cancer:*journeying toward the “unknown consequences of cancer” and the hope for freedom from cancer. Quality of life focus *b) Emotional impact of the cancer care continuum:* To overcome a personal battle with cancer and the sudden realization of the people who mattered in life. A future in flux	10/10
Raque-Bogdan et al 2018 [55] USA	Obtain a deeper understanding of how BC affects the lives of women diagnosed before the age of 40	Qualitative	13	Age—young	1. Impact of BC: a) *BC–related challenges:* e.g. fatigue, pain, anxiety, depression, or emotional exhaustion, issues with memory, concentration, or communication, challenges to family planning; *b) Coping strategies for BC–related challenges:* social support; having a choice in disclosure, provided feelings of empowerment; alternative treatment modalities; psychotherapy; avoidance/blocking it out; involvement in BC advocacy and helping others; c) *Reappraisal of self after BC; d) Reappraisal of relationships after BC* 2. Lessons learned from BC: a) *Changes in personal views; b) Positive changes in behaviour* 3. Thoughts about the future: *a) Thoughts about possibility of recurrence:* Worries or anger when thinking about recurrence; *b) Thoughts about future roles:* Family, partner, or children will be more primary; Community involvement and/or advocacy, leisure will be more important	10/10
Rashidi et al 2021 [104] Australia	Explore BC survivors’ experiences, meaning-making, and identity transformation	Qualitative	12	Survivorship experience	Sense of self: 1. Diseased Self: emotional distress, fear and uncertainty and the facade maintained before others 2. Coping Self: resilience and posttraumatic growth, epiphanies, desire to help others, optimism and positivity, psychological ownership, social support, survivorship, and empowerment 3. Transformed Self: meaning-making, psychological ownership (sense of self and ownership over the disease), empowerment, and control, losing part of one’s identity, self-disparaging thoughts and feelings of humiliation and the fear of being treated as a person with a disability, fear of stigmatization in public settings	8/10 (D, F)
Rees 2018 [56] UK	Investigate the meaning of the term “survivor” for young women; to illustrate the different meaning of invoking the survivor identity versus having the label applied to one- self by others	Qualitative	20	Survivorship identitiy Age—young	1. Accepting the survivor identity: Minority view, a positive statement about survival, albeit tentative; 2. Rejecting the survival identity: majority view, felt they could not live up to survivor image because it implied they had control over their lives and bodies. Women who did not have chemotherapy felt they had not suffered enough to earn the label. Others felt they were too young to be in the "survivor" club; 3. Cancer's ongoing presence: Women felt they would be tempting fate to call themselves a survivor, as they still experienced significant fear of cancer recurrence. They felt unable to communicate these to some key others because of expectations imposed upon the young women about cancer survivorship. This left them feeling isolated and disempowered	9/10 (F)
Schwartz & von Glascoe 2021 [110] USA (at the USA-Mexico border)	Explore what is it like to have BCin women who have had a lumpectomy or lost one or both breasts	Qualitative	8	Body image	1. Life on the border: accessing US care because it is better but without English skills and as a rural community, delayed diagnosis and treatment; 2. Liminality and life disrupted: Chronic illness disrupts the structure of everyday life, a state of ambiguity and uncertainty, no longer sick, but neither are they well, uncertian future. Fear of recurrence prevents the return to a “normal” sense of self and identity; 3. The woman in the mirror – body image and stigma: Mutilation, shame, loss of sensuality, but some did not mind the loss of breast; 4. Biographical renewal: Many women became active in advocacy for greater awareness of BC, tried to find new meaning in life, new identity	9/10 (A)
Sengun Inan et al 2020 [64] Turkey	Describe experiences of Turkish BC survivors about return to and maintenance of employment	Qualitative	12	Return to work	1. Decision-making for return to work: a) Uncertainty: questioning and doubts about personal competencies and colleagues (how they would talk and behave); b) Facilitation the value of their job, support from their colleagues during their treatment, and their doctors’ approval were facilitators for return to work; 2. Difficulties in work life: a) Burden of symptoms; b) inability to modify lifestyle to maintain health while return to work; and c) negative attitudes of employers and colleagues—having to tell employers and colleagues about BC and listening to others talking about cancer in the work environment were difficulties; 3. Sources of motivation for maintenance of work life: a) familial support; b) having a supportive workplace atmosphere (flexible working hours, sharing responsibilities and workload, having paid leaves at times of follow- ups, and lack of discriminating attitudes; c) what cancer has taught. (greater confidence and determination to live life fully and advocate for self); 4. Benefits of returning to work: a) psychological improvement (mood, feeling of competence and confidence); b) Socialisation	9/10 (F)
Sengun Inan & Ustun 2019 [92] Turkey	Explore Turkish BC survivors’ experiences related to fear of recurrence	Qualitative	12	Fear of recurrence	1. Fear of recurrence severe and linked to other related fears: e.g. of children developing cancer, of having to go through chemo again); 2. Triggered by many things: hearing cancer discussed, side-effects, follow-ups, persistent hormone therapy, self-examination, others' comments, life stressors; 3. Effects on life: Physical, emotional and social; 4. Coping: strategies focusing on feelings and thoughts, behavioural coping strategies, and social coping strategies	9/10 (F)
Son et al 2020 [76] South Korea	Understand and describe the subjective experience of cancer-related fatigue among BC survivors in-depth	Qualitative	14	Cancer-related fatigue	Impact of cancer-related fatigue 1. Traces of fighting cancer: Marker of cancer patients, negligence of the medical staff regarding the symptoms of fatigue 2. Inseparable part of the self: Shadows all around, the body being dragged 3. Difficulty in daily life: Bothersome sexual relationships, the role of the mother being diminished, social activities becoming passive 4. The body’s signals for care: Yellow traffic light, struggle for recovery	9/10 (D)
Stalsberg et al 2019 [80] Norway	a) Identify levels of-, daily routines for- and experiences with physical activity among long-term BC survivors, in general and on the part of socio-economic status groups, and b) explore whether a mixed method approach might unveil diversities of PA practice in BC survivors across SES groups	Qualitative	37	Physical activity Socio-economic status	Walking was the preferred physical activity, albeit stories about alternative activities were told Six additional themes: 1. Positive associations to physical activity 2. Fulfilling ambitions or not 3.Physical activity constraints 4. The art of balancing duties and leisure time activities 5. To appear physically active 6. Strategies for physical activity	7/10 (C, F, H)
Sun et al 2020 [65] USA	Explore the ways that lymphoedema affects BC survivors' work experience	Qualitative	13	Lymphoedema Return to work	1. BC–related lymphoedema affects physical and emotional functioning associated with work; 2. Ongoing treatment for BC–related lymphoedema creates challenges for work; 3. Environmental factors affect the return-to-work experience; 4. Personal factors play a key role in adjusting to return-to-work	9/10 (F)
Tat et al 2018 [79] USA	Qualitatively explore the sexual health experiences among racially diverse BC survivors	Qualitative	135	Sexual health Racially diverse survivors	1. Adapting to the Physical and Emotional Traumas of BC: BC survivors had to adapt to the physical and emotional traumas that resulted from surgery and treatment 2. Navigating Sexual Communication: due to changes in the body, communication about sex with significant others or potential partners was complicated and thus required considerable navigation 3. Negotiating Intimacy and Closeness without Sexual Intercourse: survivors negotiated a closeness and intimacy without sexual intercourse in their existing relationships	7/10 (D, F, G)
Toledo et al 2021 [94] USA	Explore how religion and/or spirituality influence women’s psychosocial adjustment to BC and subsequent symptom management among women on active adjuvant endocrine therpay	Qualitative	19	Religion and spirituality Adjuvant endocrine therpay	1. The psychosocial adjustment to BC: Religion and spirituality supported women in their psychosocial adjustment to BC by offering them a sense of purpose and meaning in life 2. Subsequent continued use and management of side-effects from adjuvant endocrine therapy: Religion and spirituality helped women make sense of their adjuvant endocrine therapy treatment as they persisted with it despite experiencing adverse side effects	9/10 (F)
van Maarschalkerweerd et al 2020 [66] Netherlands	Get a more detailed and comprehensive view on experiences of BC survivors regarding change in employment status 5–10 years after diagnosis, and to identify perceived barriers and facilitators regarding return to work and retaining work, both in the short and the long term	Qualitative	19	Return to work	1. Experiences regarding change in employment status 2. Barriers for return to work after diagnosis and at present. 3. Facilitators for return to work after diagnosis and at present 4. Control of barriers and facilitators which influenced (return to) work 5. Meaning of work 6. Social support of stakeholders and their involvement in the process of return to or retaining work 7. Participation in a supportive intervention program 8. Opinion about a potential supportive work-related intervention	9/10 (F)
Wilson 2020 [57] Ireland	Explore the experiences of young women who had been diagnosed with early stage BC as they transitioned from treatment to survivorship. What are the psychosocial issues of young women finishing treatment for early stage BC?	Qualitative	25	Age—young	1. A year out of your life: a sense of lost time; 2. Making changes and creating meaning: clearer priorities, appreciation; 3. Living with fear: fear of cancer recurrence; 4. Who am I now and who will I be in the future? Changing identities that were characterised by pathology and loss	9/10 (F)
Yan et al 2019 [50] USA	Examine strength‐ and culture‐related factors associated with African American female BC survivors’ cancer coping and post‐treatment experiences and make recommendations for culturally sensitive intervention	Qualitative	40	Culture Survivorship experience	1. God enables BC survivorship and works every day in our lives: a) god maintains constant protective presence; b) go engages in personal interaction and practical intervention; c) god steers every individual's life on a meaningful course; d) god makes the ultimate decision 2. The healthiest thing about us is that we are strong African American women: a) strength is made possible by god; b) family is at the core of African American women's strength; c) strength necessitates resolute positivity and unflagging momentum; d) black women can help each other be strong	9/10 (F)
Zomkowski et al 2019 [67] Brazil	Assess the barriers and facilitators experienced and the coping strategies adopted by Brazilian women 30 days after return to work following BC treatment	Qualitative	12	Return to work	1. Return to work experience: was considered a positive experience, enjoyable 2. Barriers to return to work: physical difficulties e.g. arm difficulties and fatigue, and work environment e.g. lack of social support, overprotection of colleagues and discrimination from employer 3. Facilitators of return to work: social and emotional support, positive attitudes from colleagues, cognitive demand of job vs physical demand 4. Coping strategies: work adjustment e.g. less working hours, work from home, lower workload	9/10 (F)

*BC* breast cancer. Quality rating score explanation (paper lost marks on the following criteria):

A: Was there a clear statement of the aims of the research?

B: Is a qualitative methodology appropriate?

C: Was the research design appropriate to address the aims of the research?

D: Was the recruitment strategy appropriate to the aims of the research?

E: Was the data collected in a way that addressed the research issue?

F: Has the relationship between researcher and participants been adequately considered?

G: Have ethical issues been taken into consideration?

H: Was the data analysis sufficiently rigorous?

I: Is there a clear statement of findings?

J: How valuable is the research?

### Data synthesis and interpretation

Thematic synthesis was conducted by RK, in consultation with the broader research team, whereby iterative revisions of themes and categories were discussed until consensus was reached. A meta-ethnographic approach [18] was used, including (1) extraction of findings from included reviews/papers, with an accompanying quote; (2) development of categories for findings where there are at least two examples; and (3) development of higher order categories. Categorisation involved repeated, detailed examination of assembled findings for similarity in meaning.

## Results

**Fig. 2:**
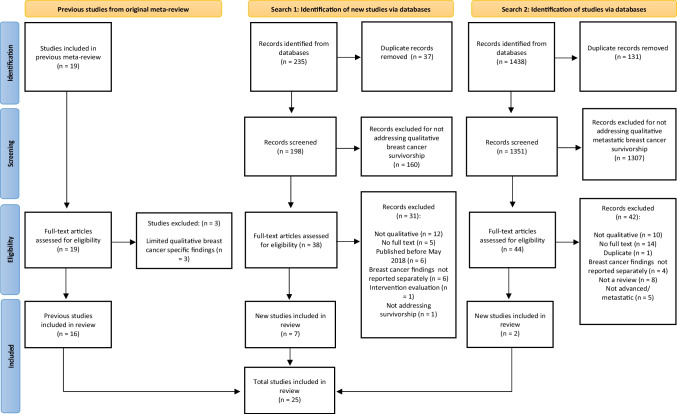
PRISMA flow chart for the meta-review literature search

Figure 2 describes the study selection process guided by the Preferred Reporting Items for Systematic Reviews and Meta-Analyses (PRISMA) [19]. Three of the 19 Laidsaar-Powell et al. [15] BC reviews were excluded as they contained very limited qualitative BC findings. After deletion of duplicates and eligibility screening, 25 systematic reviews were included in this meta-review (including 7 from Search 1 and 2 from Search 2), with an additional 76 primary papers.

### Study characteristics

**Fig. 3:**
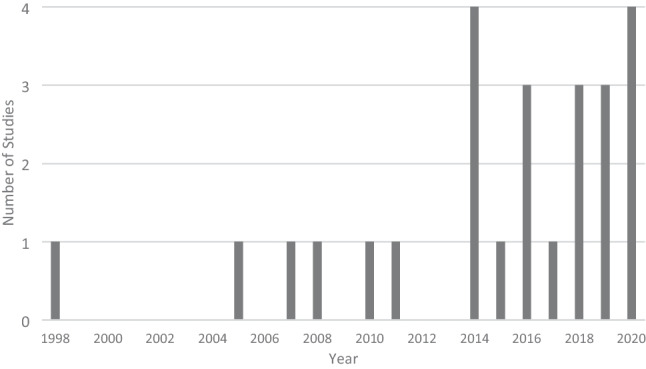
Number of systematic reviews included by year published

Eight systematic reviews included qualitative studies only and 17 included mixed methods studies. Fourteen (56%) reviews were published in the past five years (2016–2020), reflecting the recent increase in BC survivorship research (see Fig. 3). Of the papers, only three were mixed methods.

Three systematic reviews focused completely or partially on metastatic/advanced BC [5, 20, 21]. Five reviews focused on ethnic minorities including African American [14, 22, 23], Asian American [24], and Korean American [25] women. Two reviews focused on rural BC survivors [26, 27] and three focused completely or partially on age (i.e. younger and older BC survivors) [28–30]. Some reviews had a broad focus on BC survivorship and psychosocial needs, while others covered specific topics including return to work [13, 31–34], cognitive difficulties [34, 35], adherence to adjuvant endocrine therapy [36], pain [37], spirituality [20], parenthood [38], and sexual functioning [29].

Seven papers focused on metastatic BC [39–45]. Five focused on African American survivors [46–50], eleven on ‘younger’ survivors (e.g. under 50 years) [41, 43, 49, 51–58], nine on return to work [59–67], two on cognitive difficulties [68, 69], four on lymphoedema [65, 70–72], four on cancer-related fatigue [73–76], five on sexual and/or reproductive health [51, 58, 77–79], and four on healthy lifestyle factors (i.e. nutrition and exercise) [45, 54, 80, 81]. Other populations/topics explored included low SES survivors [39, 82], sexuality and gender diverse survivors [83], infant feeding [84], healthcare experience [85–87], economic burden [71], parenthood [88], posttraumatic growth [89], fear of recurrence [90–92], adjuvant endocrine therapy persistence/management [93, 94], and religion/spirituality [94]. Other papers had a wider scope, exploring BC survivors’ experiences and psychosocial needs.

### Quality assessment

See Tables 1 and 2 for an overview of the methodological quality of included reviews/papers. Six systematic reviews received a score of 10/10, indicating high methodological quality. Most reviews (12) scored in the moderate quality range (7–9/10). Seven SRs scored 5–6/10, indicating limited methodological quality. Items F (‘was critical appraisal conducted by two or more reviewers independently?’) and G (‘were there methods to minimise errors in data extraction?’) were the two most unmet items.

Fourteen papers received a score of 10/10. Most papers (60) scored in the moderate quality range (7–9/10). Two papers scored 4–6/10, indicating limited methodological quality. Item F (‘has the relationship between researcher and participants been adequately considered?’) was the most unmet item.

### Thematic analysis

From the included reviews/papers, 8 over-arching themes were identified: (1) Ongoing impact and search for normalcy, (2) Uncertainty, (3) Identity: Loss and change, (4) Isolation and being misunderstood, (5) Posttraumatic growth, (6) Return to work, (7) Quality of care, and (8) Support needs and coping strategies. These themes are presented with subthemes and participant quotes selected from systematic reviews (reviews) and primary papers (papers), where appropriate.

## Theme 1: Ongoing impact and search for normalcy

### “Am I healthy or am I not”?

The ongoing impact of BC and its treatment came as a shock to many women as they transitioned into survivorship [36]. Many systematic reviews examined the impact of ongoing symptoms on survivorship, including persistent pain, fatigue and weakness, feeling unwell, sleeping difficulties, lymphoedema, impaired cognition (including ‘chemobrain’), skin conditions, menopausal symptoms, sexual problems, and fertility issues [5, 13, 21, 22, 29, 33–37], as did primary papers (PPs) [41, 44, 49, 51, 52, 55, 68–70, 73–76, 79, 84, 85, 95–102]. These ongoing side-effects challenged expectations that after treatment, BC survivors would return to their premorbid level of health. Instead, survivors found themselves in a space between illness and health [Review: [37]].

Now I've finished my treatment but am stuck in a period where I sit and think ‘am I healthy or am I not?’ It's like something in between [Review: [37]]

You kind of think, you’ll have your surgery…and then life will go back to normal, but it doesn't [PP: [85]]

Physical and cognitive symptoms led to significant limitations that impacted daily life (e.g. inability to clean or drive), as well as impairing social, occupational, and physical activities [Reviews: [5, 13, 21, 33, 34, 36, 37]; Papers [44, 70, 95, 96]].

*I can't ride a scooter, I can't raise my arms… I can't lift a pack of milk, it's too painful [Review: *[37]*]*

Less able to play with grandkids or do simple chores requiring strength or lifting [Paper: [70]]

Some survivors reported feelings of desperation and fatalism that their symptoms would never improve [Review: [37]].

Sometimes when I wake up I think ‘will the pain be like this every day, always, always…’ that's hard to manage sometimes [Review: [37]]

Due to the significant impact of persistent side-effects, some survivors began to value their quality of life (QOL) (i.e. symptom management) over length of life [Review: [36]]. For example, some BC survivors chose to cease adjuvant treatment designed to prevent recurrence and prolong life, to reduce debilitating side-effects and increase quality of life [Review: [36]; Paper: [93]].

*I said, I’ve had tamoxifen, and I’ve had breast cancer. I would rather have breast cancer [Review: *[36]*]*

I chose a lesser time left. I said at my age, does it matter if the cancer comes back one way or another… I like my home and I like being involved in the community, going to the club and that. Coming off the tablet has given me back that quality of life [Review: [36]].

### Psychological impact

BC survivors also experienced psychological problems due to enduring side-effects, ongoing uncertainty and concern about the future. They expressed feelings of sadness, shock, guilt, insecurity, worry, anger, fear, disappointment, distress, and grief [Reviews: [5, 21, 28, 29, 33]; Papers: [41, 49, 52, 55, 57, 68, 70, 74, 75, 79, 96–99, 102–104]], especially those experiencing recurrence [Review [5]:].

It [BC recurrence] was such a dreadful disappointment that I got a feeling that it didn’t matter what I did. That little devil who has sunk his claws into me isn’t going to let go… the disappointment was enormous [Review [5]:]

### “Things will never be normal, and that’s awful”

Many survivors sought normalcy after treatment: a return to pre-cancer health and ability [Reviews: [5, 13, 21, 22, 28, 36]; Papers: [95, 97, 100, 105, 106]]. Re-establishing normalcy involved adjusting daily activities to match limitations, focusing on relationships, and not focusing on the BC [Review: [21]; Paper: [69]]. Additionally, women attempted to rebuild their pre-treatment lives (and identity) to re-establish normalcy, including returning to work [Review: [13]]. For some, a return to normalcy was perceived as impossible. Significant changes to the body (e.g. scarring, menopausal symptoms, physical and cognitive impairment) and to lives (e.g. career and relational disruptions) made the life that women once had, or imagined for their future, impossible to reach [Review: [28]; Papers: [70, 107]. Recurrence exacerbated this sense of loss [Review: [5]].

I feel so terribly sad that things will never be normal again. Things will never be normal, and that's awful [Review [5]:]

This [BC related lymphoedema] will affect my life forever [Paper: [70]]

## Theme 2: Uncertainty

### Patient to survivor

BC survivors underwent a transition from undergoing primary treatment (being a patient) to life post-treatment (survivorship) and this transition was characterised by uncertainty regarding future quality and length of life [Reviews: [28, 37, 108]; Papers: [91, 96, 109, 110]]. This uncertainty was exacerbated by decreased support from and contact with health providers and other patients, as women shifted to self-management while losing the hospital ‘safety net’ [Reviews: [14, 37, 108]; Papers [60, 85, 96, 100]:].

The problems start after that [end of treatment]: whom do you turn to when you have pain in your hip like I do? [Review: [37]]

### Uncertainty of symptoms

BC survivors experienced uncertainty and worry related to ongoing symptoms and need for further treatment (e.g. ongoing adjuvant treatment) [Reviews: [34, 36, 108]; Papers: [48, 109]]. One review noted survivors were unsure about the likely duration of ongoing cognitive impairment, which was compounded by a lack of information about their symptoms [Review: [34]].

### Fear of recurrence and death anxiety

The transition into survivorship and loss of regular contact with the treatment team heightened many women’s fears of cancer recurrence (FCR) [Review: [14]]. FCR, recently defined as “fear, worry or concern relating to the possibility that cancer will come back or progress” (p. 3266)[111], was a common and significant issue for survivors [Reviews: [28, 30, 36–38, 108]; Papers: [52, 55–57, 82, 90–92, 99, 101, 109, 110, 112]]. FCR focused women on the uncertainty of their future [Review: [5]].

One of my biggest fears is the 5-year waiting period, to find out if we are going to survive or not. That creates suspense, fear, and negative emotions… I feel like I’m standing on a balance just waiting to see which way it is going to go [Review: [5]].

The furthest I can think is the coming weeks and months. I don't make long-term plans [Review: [5]]

FCR can lead to excessive vigilance regarding symptoms. While some degree of symptom monitoring is required for early detection of recurrence if it occurs, hypervigilance can lead to ongoing and exacerbated fears and anxiety. Many women had difficulty determining what is ‘normal’ and what may be a sign of recurrence [Reviews: [37, 108]; Paper: [52]].

You really listen to your body in quite a different way now. Every little thing you feel in your body could be signs of something abnormal [Review: [37]]

Women with metastatic BC were particularly hyper-vigilant while monitoring for signs of disease progression [Reviews: [5, 21]]. The need for symptom monitoring and the possibility of recurrence meant the survivorship period had no certain endpoint [Review: [108]].

### “My time’s running out”

Reviews found a preoccupation with death in BC survivors [Reviews: [28, 37]], particularly in survivors with advanced BC [Reviews: [5, 20, 21]]. For some women, this led to a sense of urgency to live life to the full, leaving them out of step with friends and family [Review: [37]; Paper: [41]].

It felt like everyone was driving too slowly and I didn't have the time to sit there and wait… I felt like ‘you have all the time in the world, but my time's running out’ [Review: [37]].

Younger women coped with thoughts of death by attempting to have some control over the process through communicating their dying wishes, while mothers coped by making plans to ensure their children would be well cared for [Review: [28]]. However, not everyone feared death, as was found for some survivors with advanced BC [Review: [5]].

I've kind of come to terms with these fears, and I'm not really afraid of dying [Review: [5]]

## Theme 3: Identity: loss and change

### “I’m different… I’m imperfect”

Bodily changes due to BC and its treatment (including loss of hair and one or both breasts), meant that many women were persistently reminded of their cancer due to an altered body image. Some felt a loss of control and alienated from their bodies, disfigured and undesirable, with a changed identity [Reviews: [5, 14, 21, 22, 28, 29, 113]; Papers: [48, 51, 53, 70, 78, 95, 98, 99, 101, 103, 104, 110, 112, 114]]. However, some survivors did not experience body image disturbances or were able to adapt to changes over time [Papers: [53, 110]], even viewing their scars as positive signs they were disease-free [Review: [113]].

Each time I passed a mirror I jumped back because I didn't recognise myself [Review: [5]]

I'm different from those who are normal… For myself, I'm imperfect. I had the surgery and lost one side [of the breast] [Review: [113]]

I saw myself and I felt bad they had taken my breast. But then, I said, `No. Thank God. Because it was taken, I live.' Then I was assimilating, and now it's normal for me, that I don't have my chest [Paper: [53]].

For some women, losing a breast led to changes in their sense of femininity and womanhood *[Review: *[113]*].*

… you started discovering that you were now just half a woman ‐ my femininity disappeared… [Review: [113]]

In reviews focusing on African American BC survivors, hair loss from treatment (and changes to hair texture and colour), as well as body altering surgeries, left women feeling damaged and less feminine and this was amplified by a desire to appear strong and to look well [Reviews: [14, 22]].

### Sexuality and relationships: “I felt something was missing”

The impact of BC treatment on women’s bodies also affected sexuality and intimate relationships [Reviews: [5, 21, 22, 29, 113]; Papers: [53, 58, 76–79, 95–97]]. Insecurities after mastectomy reduced women’s sense of sexual desirability [Reviews: [5, 22, 113]; Paper: [77]].

The majority of us feel degraded as women as we see ourselves in the mirror and wonder, ‘If we cannot accept ourselves, how can our husbands or partners?’ [Review: [5]]

Other treatment side-effects impacting sexual activity and intimacy included early menopause, pain, vaginal dryness, and reduced sexual desire/libido [Reviews: [22, 29]]. Some women felt rejected by partners due to changes in their relationship [Review: [22]].

### Fertility and infertility

Reviews/papers examined association between identity and fertility and how BC and treatments posed a threat to this part of women’s lives [Reviews: [28, 29, 38]; Papers: [52, 53, 88, 96]]. For some women, especially pre-menopausal women, loss and grief related to infertility was significant and served as an emotional reminder of BC [Review: [28]]. For other survivors, a pregnancy post-cancer was considered restorative and normalising [Paper: [84]].

To have something grow inside you on purpose in contrast to this cancer that grew unwelcome… you’re trusting in your body again…It felt just so normal [Paper: [84]]

Fertility, for others, was viewed as secondary to survival and preventing recurrence [Review: [38]]. While some women desired children in the future, others decided against children due to FCR, genetic risk and the health of the baby [Review: [38]].

### Changing and maintaining roles

For many survivors, there was a significant shift in roles and relationships, from providing to receiving care [Reviews: [22, 25, 28, 108]; Paper: [76]]. There was also a desire (and sometimes expectation from others) to maintain identity and normalcy by fulfilling former roles, such as upholding the role of homemaker [Review: [34]], returning to work [Review: [13]], or caregiving [Reviews: [25, 28]; Papers: [39, 43, 114]]. For some women, however, this expectation to fulfil their pre-diagnosis role was a challenge and burden [Review: [25]].

Even after getting chemo, I still had to take care of my children, so that was hard [Review: [25]]

## Theme 4: Isolation and being misunderstood

Limitations and stigmatisation meant some survivors isolated themselves or felt unable to fully participate in social life, consequently reducing the social support available to them [Reviews: [21, 113]; Papers: [44, 48, 72, 74, 90, 103]]. Some survivors, especially older survivors, reported not wanting to burden those around them [Reviews: [25, 28]; Paper: [90]]. Many also felt misunderstood by others (including family, community and co-workers) as they struggled with ongoing challenges from their diagnosis and treatment. Survivors described an unrealistic expectation from those around them that they would fully recover and be symptom-free after primary treatment [Reviews: [13, 14, 25, 32, 34, 37]; Papers: [56, 107]], especially when they may appear physically well to others [Paper: [43]].

Families don’t understand. They say they understand, but they expect us to be the same people as before the disease [Review: [25]]

That’s one of the things that people don’t understand about having stage IV cancer. I think a lot of people think [your] appearance should be bald and super thin and kind of sickly looking. When I tell people, “I’ll always have stage IV cancer,” they look at me. “No, you don’t.” I’m like, “I look normal, I know.” You can look normal. People don’t realise that [Paper: [43]].

## Theme 5: Posttraumatic growth

### “To grow from it, to heal from it”

Many reviews/papers identified posttraumatic growth alongside the negative impacts of BC and its treatment [Reviews: [5, 13, 21, 28, 32, 34, 113]; Papers: [39, 48, 55–57, 89, 102, 104, 105, 109, 110, 114]]. Some women were able to embrace and accept their new bodies [Reviews: [5, 113]; Paper: [114]].

[Cancer] definitely changed my life… for the better. It gave me more of a clarity about myself… to take it and grow from it, and heal from it, and achieve from it [Paper [39]:]

I like my body better now… I've accepted it so I like my body… it's part of life you know and you just get on with it… [Review: [113]]

*‘Wakeup call’*

Some survivors also reported a greater appreciation for life and a sense of gratitude, viewing their cancer experience as a turning point and making the most of their lives now [Reviews: [5, 21, 28, 34, 113]; Papers: [39, 57, 102]].

Little things now mean a lot to me. I don't take life for granted any more [Review: [5]]

[Cancer] definitely changed my life, but it changed it for the better. It gave me more of a clarity about myself [Paper [39]:]

For some, the shift to focusing on appreciating life included a re-evaluation of their work/life balance, [Reviews: [13, 32]; Paper: [55]], working towards personal goals and values, prioritising relationships, and contributing to the community [Reviews: [21, 34]; Papers: [48, 89, 109]]. This included a sense of empowerment through focusing on healthy lifestyle changes and self-management [Papers: [48, 105, 109]]. Finding meaning in the BC experience was realised by connecting with (and supporting) others diagnosed with BC and advocating for increased BC awareness [Reviews: [5, 20, 21, 28]; Papers: [48, 55, 109, 110]].

## Theme 6: Return to work

Four reviews specifically focused on the return-to-work experience for BC survivors [Reviews: [13, 31–33]], while one review discussed return to work in the context of cognitive changes [Review: [34]]. Employment was also discussed in many primary papers [Papers: [53, 59–68, 95, 99]]. Returning to work was important to many survivors as a way of regaining a sense of normalcy, meaning, identity, support and connection [Reviews: [13, 32, 34]; Paper: [95]]. However, some survivors found the work environment unsupportive, with some even facing discrimination [Reviews: [31, 32]; Papers: [53, 63]]. Privacy regarding disclosure of illness was an issue; some survivors found that employers did not keep their health status confidential [Review: [32]]. Treatment side effects and body insecurities led to challenges and loss of confidence at work [Reviews: [13, 31, 33]; Papers: [63, 64]].

I had to lean down to do anything on the bottom, lower shelf or even for bags to pack them, I was like this [covered her chest] all the time, holding it together… every minute of my working day you’re thinking of it [Review: [33]].

Some survivors reported cognitive impairments such as problems with concentration, executive function, memory, and speed of processing [Review: [33, 34]; Papers: [63, 68]].

With this memory thing, I was very frustrated at work and so I thought that I can’t go on like this. It was a chore now going to work than a joy [Review: [33]]

Many survivors experienced anxiety and frustration around their capacity to return to work [Reviews: [13, 31–33]]. This was further complicated by employers expecting survivors to be as capable post-treatment as they were pre-diagnosis and survivors not wanting to disappoint or mislead them [Reviews: [13, 31, 32]]. Survivors also described financial pressure to return to work [Reviews: [13, 32]].

## Theme 7: Quality of care

### Health care experiences

Many reviews focused on experience of the health care system as a cancer survivor. While many women reported positive healthcare experiences, some reported negative interactions. AlOmeir et al. [36] reviewed factors influencing survivors’ adherence to adjuvant treatment, finding that the decision to accept or delay treatment was influenced by trust in health care providers as well as concerns, expectations and knowledge of the treatment. Selamat et al.’s [34] review, focused on experience of cognitive changes, found survivors experienced a lack of information about cognitive deficits and felt invalidated and dismissed by health professionals. Some survivors with metastatic BC reported that their needs were not met if care focused on physical symptoms to the exclusion of psychosocial needs [Review: [21]].

### Feeling alone; lack of information and support

Survivors noted a need for information about the reality of survivorship and disease management, especially about ongoing side-effects [Reviews: [22, 28, 30, 31, 34, 36, 37]; Papers: [60, 87, 95, 98–100, 109]].

They (the doctors) said in a year you'll be back to your regular everyday life, and I'm not. It's a disappointment [Review: [37]]

We feel lost, really. There is a lot of information missing—information about knowing what to do, where to call [Paper: [60]]

Survivors also noted a need for relevant information, empathy and support from health services, otherwise they felt dismissed, unsupported and alone [Reviews: [36, 37]; Papers: [40, 46, 51, 68, 72, 82, 84, 85, 90, 91, 97, 105]].

I wished that my pain at home was followed up much more [Review: [37]]

#### Barriers

Reviews identified barriers for survivors to access health services. For rural BC survivors, location and transport needs were a barrier to care [Review: [26]]. For some low SES and/or ethnically diverse BC survivors, there were concerns about the quality of care they had access to [Paper: [39]].

It’s a county hospital, so it’s an overly stressed system … They don’t have resources … I was told that in a private institute, you were assigned a nutritionist, a social worker and a binder that had everything broken down … I wish we had a universal medical system and when you get cancer this is what you get [Paper: [39]].

Several primary papers highlighted the ongoing financial burden/barrier associated with BC due to healthcare cost and productivity loss [Papers: [82, 99, 103, 105, 112]], including BC survivors with lymphoedema [Paper: [71]].

I lost my job ‘cause I got diagnosed with breast cancer so financially it was very difficult … I was out of work for almost a year … with the chemo… I was really sick and then I went back against the doctor’s orders ‘cause I needed to make money… When I came back to work that’s when they expected me to resume all of the duties… full force and…I got fired… [Paper: [71]].

Language can also pose a significant challenge when seeking information and support. Wen et al. [Review: [24]] found for Asian American women, communication with health professionals was sometimes challenging. Similarly, survivors struggled to find support groups in their community when there was a language barrier or perceived cultural differences [Review: [25]].

Americans don’t seem to share their emotions with immigrants like us. They don’t try to talk to us first… [Review: [25]]

## Theme 8: Support needs and coping strategies

### Social support

BC survivors reported needing practical and emotional support from family, partners, friends, community groups, co-workers, other BC survivors and health providers [Reviews: [5, 14, 21–23, 25–29, 34, 36, 115]; Papers: [39, 43, 47–49, 53–55, 68, 69, 74, 78, 82, 84, 95, 97, 99, 105, 109, 112, 114, 116]]. These supports helped survivors to engage in activities, contribute to community, talk about their experiences, and cope with distress [Reviews: [21, 34]]. Support from other survivors was important due to shared experiences [Reviews: [21, 22]; Papers: [69, 73, 84]].

My kids are my all and being with them keeps me going. Even through what I’m going through now.. They’re like my sun. I see them, and I light up [Paper: [39]]

Yeah when I met fellow survivors at BCF (Breast Cancer Foundation) … yeah … I thought, they also experienced what I have experienced. So it’s OK. It’s not too bad and we laughed about it [Paper: [69]].

### Spirituality

Spirituality was important to many survivors in coping with their BC and its ongoing impact on their lives [Reviews: [5, 14, 20–24, 27, 29, 115]; Papers: [39, 41, 47–50, 78, 94, 97, 102, 103, 109, 114]], as it helped them to cope with uncertainty and accept their condition [Reviews: [5, 20, 21]].

He's chosen me to survive this cancer journey. It's really helpful to me to have a higher power that I choose to call God and to believe that I have a purpose in this world [Review: [5]].

My spirituality and belief in God are so strong and my faith keeps me strong [Paper: [39]]

#### “We must learn to live with it”

Besides social support and spirituality, women found other ways to cope, including adapting daily activities to accommodate limitations, accepting side-effects and integrating the disease into current life [Reviews: [27, 37, 115]].

I learned to change some of my movements. I learned movements that relieve. Instead of wringing the kitchen glove like that, now I wring it like this, against the side of the sink [Review: [37]].

Others, especially women with advanced BC [Reviews: [5, 21, 22]], and rural BC survivors [Review: [27]], coped through avoidance or denial of their disease, such as trying to forget about their condition [Paper: [90]].

Thinking positively and hopefully, as well as having a ‘fighting spirit’ towards the disease and its ongoing impact, was important for some [Reviews: [5, 21–23, 115]; Paperss: [41, 47, 107]].

I believe that a person should be satisfied and not embittered…. Constant anger will cause more disease [Review: [5]]

Many older survivors coped by not having the expectation they would return to full health, as they had already begun to accept declining health as part of aging [Review: [28]]. These survivors focused on their present experience rather than focusing on the past or future [Review: [28]].

## Discussion

This meta-review identified and synthesised 25 systematic reviews, and an additional 76 primary papers to describe the psychosocial experience of BC survivors. Overall, the quality of included reviews and papers was mixed, with the majority of studies classified as of moderate quality, suggesting that future research could be improved by following recommended methodological procedures [11]. Some of the included reviews/papers focused on specific groups of BC survivors, including younger/older, rural, ethnic minorities, and survivors with metastatic BC. Return to work was well covered, as was quality of care. Ongoing symptoms (e.g. physical, cognitive, psychological and sexual) was an area of saturation within the included systematic reviews.

Eight of the included systematic reviews were published after the Laidsaar-Powell et al. [15] meta-review and focused on adherence to adjuvant endocrine treatment, rural BC survivors, pain, return to work, sexual problems, metastatic BC, transitioning from patient to survivor, and survivorship characteristics of different BC stages (post-treatment to recurrence and metastatic BC). More recent reviews emphasised ongoing physical and psychological impacts, changes to identity and roles, as well as the transition from patient to survivor. In recent papers, similar topics were covered with many papers focusing on specific populations (e.g. metastatic BC, BC survivors with lymphoedema, low SES, African-American, sexual and gender diverse, young BC survivors) and/or topics (e.g. sexual and reproductive health, cognitive impairment, return to work, posttraumatic growth, cancer-related fatigue).

After combining all of the reviews/papers, the meta-review synthesis resulted in eight overarching themes. A key thread across many of these themes was the challenging transition from patient to survivor; characterised by searching for normalcy, ongoing treatment and side effect impacts, feelings of uncertainty, FCR, lack of information, feeling misunderstood, and changing roles and identity. For many women, this transition was complicated by unmet expectations that once treatment was completed women would return to their healthy premorbid life, which was not a reality for many. Instead, they faced ongoing symptoms and limitations, which in turn led to a sense of being misunderstood by family members and employers. For those women who belonged to an ethnic minority group, challenges could be further exacerbated by difficulties communicating with health professionals and accessing support from within their own communities [24, 25].

These themes reflect the findings of a scoping review by Maheu et al. [117] which focused on uncertainty and FCR in BC survivors. The authors found that the experience of uncertainty was characterised by doubt, liminality, a sense of insecurity, and an inability to meet expectations. Uncertainty was also found to be a cause of FCR. Lack of information and lost connection to health professionals were found to exacerbate both uncertainty and fear of recurrence [117], as was noted in the current study.

While most research reported in this review focused on the ongoing challenges faced by BC survivors, within some reviews women also reported positive aspects of their survivorship experience, including greater appreciation of life. Positive outcomes have been previously identified in survivorship research, emphasising the resilience demonstrated by many cancer survivors [118]. Posttraumatic growth refers to the positive change (or growth) that occurs in individuals after a significant stressor [119, 120]. Reflecting these domains, many cancer survivors report appreciation for the value of life, more self-confidence and self-esteem, positive social interactions and stronger interpersonal relationships, reprioritisation of personal values, as well as strengthened religious faith and spirituality [118, 119] after BC.

### Clinical implications

Due to the long-lasting impacts that BC survivors face during their survivorship, highlighted in this review, these women require ongoing support to manage their symptoms and improve both quality of life and metastatic BC survival outcomes [4, 121]. BC survivorship research can inform psychosocial interventions and these should ideally be embedded into established patient care [122]. Interventions may include individualised support, with regular assessment of ongoing challenges and survivorship needs, as well as addressing healthy lifestyle changes, treatment adherence, and symptoms where needed [123]. Support during survivorship should be sustained and more than a one-off consultation [123]. For rural BC survivors, scheduling all appointments on one day and providing support and information via various sources (e.g. social media, internet resources) are suggested ways of improving their health care experience [26].

One important strategy to address the long-term needs of BC survivors is the use of survivorship care plans (SCPs) which are designed to provide support and information during the transition into survivorship [124]. Individualised SCPs created by the oncology treatment team [124] can provide information regarding quality of life and concerns for BC survivors, as well as including plans for follow-up and disease recurrence surveillance [124]. SCPs aim to provide BC survivors with a realistic understanding of what to expect as they transition into survivorship, normalising their feelings of uncertainty. Kozul et al. [125] noted that SCPs can promote discussion of varied survivorship issues, including side effects, FCR, medication adherence, psychosocial/mental health, bone health, difficulties with relationships, exercise, and fertility, and prompt referral to appropriate health professionals for help with these issues.

Interventions aimed specifically at body image, sexuality, and identity may also help women struggling with the impact of BC on their bodies (e.g. after mastectomy) and function (e.g. inability to return to work/care). Morales-Sánchez et al. [126] systematically reviewed eight studies of interventions aimed at improving body image and self-esteem, finding varied effectiveness. Of the interventions, group therapies (e.g. cognitive behavioural therapy groups) were found to demonstrate the most positive results. Other interventions, such as psychosexual counselling and intimacy enhancement (couple-based) interventions, have also been found to improve sexuality and intimacy concerns [127, 128].

An important consideration for all interventions is cultural appropriateness. As highlighted in our findings, many women prefer to access support that includes others like them, conforms with their values and beliefs, and which can help them with specific issues such as challenges with language and communication.

### Further research

While the current review highlights the large body of evidence examining BC survivorship, several gaps in the evidence base are noted, including BC survivors with *BRCA*1/2 gene mutations, women receiving tailored treatments (including emerging treatments such as immunotherapy/targeted therapies), those of low socioeconomic status, and the unique experiences of BC survivors with multimorbidity and complex health care needs. While the included reviews discussed the impact of various long-term side-effects of BC and its treatment (e.g. sexual dysfunction, cognitive impairment, psychological distress, loss of fertility, sleep impairment, body image concerns, lymphoedema, ongoing pain, and fatigue), some late effects (emerging months or years after treatment completion) were not covered, including cardiotoxicity and osteoporosis [123, 129]. Lifestyle changes are recommended to reduce risk of cardiotoxicity and osteoporosis including addressing tobacco and alcohol use, managing weight, and increasing exercise [123, 129]. These lifestyle changes may also decrease the risk of recurrence and are therefore commonly part of BC survivorship care [121, 123, 124, 129]. Despite the importance of lifestyle changes in survivorship, this was rarely discussed in the included reviews/papers.

Experiences of gender and sexually diverse BC survivors were also largely missing from the included reviews/papers, reflecting a paucity of research within this area. A recent PP from Brown and McElroy [83] that focused on the unmet needs of sexual and gender minority BC survivors found that these survivors experienced a lack of appropriate support from health care systems and BC survivor organisations.

### Strengths and limitations

This study has several limitations. Searches were limited to reviews published in English and, therefore non-English reviews may not have been included. Systematic reviews may be limited by the interpretation provided by these reviews and their choice of quotations, and this review only included primary papers published after the last search made by a systematic review. Therefore, some detail may be missing. Additionally, it is likely that some included reviews drew upon the findings of the same primary papers; therefore, some duplication of results across reviews is possible.

By focusing on qualitative and non-intervention studies, this meta-review may be missing valuable information about the BC survivorship experience. Future studies could incorporate a wider range of research types. Nevertheless, this meta-review is the first to synthesise the available qualitative evidence of BC survivorship and provides the most comprehensive overview of BC survivor experiences to date.

## Conclusion

This meta-review synthesised the qualitative BC survivorship evidence base and found that BC survivorship is characterised by many ongoing physical and psychosocial impacts, as well as posttraumatic growth in some women. The quality of research within this meta-review was moderate, and future studies should make use of methodological quality guidelines when conducting systematic reviews and primary research. The findings suggest that BC survivors experience significant uncertainty and changes in identity, along with ongoing physical and psychosocial challenges. It is therefore important to provide timely and accessible support to these women, such as through the use of individualised SCPs. Specialised information about and support for ongoing effects and interventions aimed at body image, sexuality, and identity may be beneficial. To address gaps in the BC research, future studies should include BC survivors with *BRCA*1/2 gene mutations, women receiving tailored treatments, women from low socioeconomic backgrounds, BC survivors with multimorbidity and complex health care needs, late effects, as well as interventions targeting gender and sexually diverse BC survivors.

### Supplementary Information

Below is the link to the electronic supplementary material.

Supplementary file1 (DOCX 17 KB)

## Data Availability

Data are available within an institutional repository.
