# Supplementary material for: Psychosocial experiences of breast cancer survivors: a meta-review
Source: J Cancer Surviv. 2023 Mar 1;18(1):84–123. doi: 10.1007/s11764-023-01336-x (PMC10866753; doi:10.1007/s11764-023-01336-x)
Supplement: Supplementary file 1 — Supplementary file1 (DOCX 17 KB) [file 11764_2023_1336_MOESM1_ESM.docx]

**Supplementary Table 1** Joanna Briggs checklist for systematic reviews and research synthesis

| Is the review question clearly and explicitly stated? |
| --- |
| Were the inclusion criteria appropriate for the review question? |
| Was the search strategy appropriate? |
| Were the sources and resources used to search for studies adequate? |
| Were the criteria for appraising studies appropriate? |
| Was critical appraisal conducted by two or more reviewers independently? |
| Were there methods to minimise errors in data extraction? |
| Were the methods used to combine studies appropriate? |
| Were recommendations for policy and/or practice supported by the reported data? |
| Were the specific directives for new research appropriate? |

**Suplementary Table 2** Critical Appraisal Skills Programme Qualitative checklist

| Was there a clear statement of the aims of the research? |
| --- |
| Is a qualitative methodology appropriate? |
| Was the research design appropriate to address the aims of the research? |
| Was the recruitment strategy appropriate to the aims of the research? |
| Was the data collected in a way that addressed the research |
| Has the relationship between researcher and participants been adequately |
| Have ethical issues been taken into consideration? |
| Was the data analysis sufficiently rigorous? |
| Is there a clear statement of findings? |
| How valuable is the research? |
